# Retrospective analysis of arthroscopic repair outcomes using a modified Mason-Allen suture bridge “parachute” technique with bone marrow stimulation for rotator cuff tears

**DOI:** 10.1371/journal.pone.0356245

**Published:** 2026-08-20

**Authors:** Shaobo Li, Shuo Zheng, Xin Sun, Chaochao Dou

**Affiliations:** 1 Department of Sports Medicine, Qilu Hospital (Qingdao), Cheeloo College of Medicine, Shandong University, Qingdao, Shandong, China; 2 Department of Orthopedics, Qingdao Central Hospital, University of Health and Rehabilitation Sciences, Qingdao, Shandong, China; Carol Davila University of Medicine and Pharmacy: Universitatea de Medicina si Farmacie Carol Davila din Bucuresti, ROMANIA

## Abstract

**Objective:**

Rotator cuff tear (RCT) is a prevalent source of shoulder pain and functional limitation, with postoperative tendon healing continuing to present significant challenges. This study aims to retrospectively evaluate the clinical and imaging outcomes of RCT treatment at our institution, employing a modified Mason-Allen suture bridge technique in combination with bone marrow stimulation (BMS).

**Methods:**

A retrospective study was conducted involving patients with RCT who underwent arthroscopic modified Mason-Allen suture bridge repair, also referred to as the “parachute” technique, combined with BMS at our institution between January 2021 and January 2023. Exclusion criteria included preoperative magnetic resonance imaging (MRI) demonstrating Goutallier grade 3 or higher fatty infiltration, a history of shoulder surgery, the presence of degenerative arthritis, or rotator cuff lesions. A total of 47 patients met the inclusion criteria, of whom 44 completed a minimum follow-up period of 24 months. Preoperative and 24-month postoperative data were obtained from electronic medical records and outpatient follow-up systems. These data encompassed the American Shoulder and Elbow Surgeons (ASES) score, Single Assessment Numeric Evaluation (SANE), Visual Analog Scale (VAS) for pain, Japanese Orthopaedic Association (JOA) score, shoulder range of motion in flexion, external rotation, and internal rotation, as well as postoperative MRI assessments to evaluate tendon healing status.

**Results:**

Compared to preoperative measurements, patients exhibited significant improvements in VAS, SANE, ASES, and JOA scores, as well as in all ranges of motion at 24 months postoperatively (P < 0.05). In the final follow-up, MRI assessment employing the Sugaya classification demonstrated that all 44 patients (100%) exhibited healing (types I–III), with no instances of re-tear (types IV–V) observed. Throughout the study period, no instances of nerve or vascular injury, wound infection, or complications related to suture anchors were observed.

**Conclusion:**

This retrospective analysis indicates that the rotator cuff repair strategy, which integrates the “parachute” technique with BMS, results in significant functional improvement and consistent imaging-confirmed healing during follow-up, accompanied by a low incidence of complications. This retrospective analysis suggests that combining the “parachute” technique with BMS constitutes a safe and clinically viable approach for rotator cuff repair, thereby providing a basis for future prospective comparative investigations.

## 1. Introduction

Rotator cuff tears (RCT) are a common condition affecting the shoulder joint, particularly among the middle-aged and elderly populations [1–4]. This condition usually cause shoulder pain and functional limitations, which can significantly impact the daily activities of patients [5,6]. At present, a variety of surgical interventions are available for the management of RCT [4,7–9]. Although the ongoing advancements in arthroscopic repair techniques, achieving successful tendon healing continues to face significant challenges. According to previous research reports, the re-tear rate after rotator cuff repair remains between 17% and 47% [10–12]. Lee et al. reported that, in an magnetic resonance imaging (MRI) follow-up conducted six months postoperatively on 102 patients with small to medium-sized RCT, 24.4% of the patients experienced a retear [13]. Similarly, Longo et al. found that approximately 12.5% of small to medium-sized RCT and up to 37% of large RCT resulted in retears [14]. Consequently, in the context of arthroscopic surgery for RCT, optimizing surgical techniques to enhance tendon healing rates and, subsequently, improve the overall success rates of the procedure has emerged as a primary focus within clinical research.

The healing process of tendons is primarily influenced by two key factors: the strength of mechanical fixation and the degree of biological healing [15–23]. The strength of mechanical fixation is primarily influenced by the various suturing techniques employed during surgical procedures [24–26]. Currently, the modified Mason-Allen technique, when used in conjunction with the suture bridge technique, increases the contact area between the tendon and the bone by applying two rows of anchors [27–29]. This approach effectively reduces movement at the bone-tendon interface and enhances resistance to rotational forces. Although this technology provides several advantages over traditional single-row or double-row repair techniques, the rate of re-tearing has not demonstrated a significant reduction. The primary factors contributing to this observation may include the necessity for the repaired tendon to withstand increased tensile forces, the constriction of the tendon resulting from medial suturing, and further injury to the already compromised torn tendon. An alternative approach to facilitate tendon healing involves the application of biological enhancement techniques, including bone marrow stimulation (BMS) [30,31]. Studies have demonstrated that BMS can elicit the release of active constituents from the bone marrow, including bone marrow mesenchymal stem cells and growth factors [32]. These constituents contribute to the establishment of an optimal local microenvironment for tendons, thereby improving the regeneration and repair of tendon tissue. It is important to note that an increasing number of meta-analyses have substantiated the clinical efficacy of BMS in reducing postoperative retear rates. A systematic review and meta-analysis by Ajrawat et al. demonstrated that, compared to a control group without BMS, the application of BMS in conjunction with primary arthroscopic rotator cuff repair significantly decreased the retear rate [32]. Similarly, Zhang et al. confirmed that BMS is an economical, effective, and easily performed technique that substantially lowers the retear rate following arthroscopic rotator cuff repair [30]. These quantitative findings provide robust biological and clinical theoretical support for incorporating BMS into the modified suture bridge technique.

According to the strengths of the two previously mentioned methodologies, we have integrated and modified the Mason-Allen suture bridge technique with the BMS technique, aiming to harmonize the dual advantages of mechanical stabilization and biological augmentation. We have designated this novel approach as “Parachute” technology, due to the final configuration of the sutures resembling an open parachute, which effectively secures and envelops the tendon. Given the retrospective design of this study, its primary objective is not to establish the superiority of the new combination but rather to objectively assess its actual performance in routine clinical practice. Since the adoption of the “parachute” technique at our institution, we have systematically collected mid- to long-term follow-up data from consecutive cases. This study utilizes a retrospective observational approach to comprehensively analyze the postoperative functional outcomes of patients with RCT treated using this modified procedure. Outcome measures include pain scores (VAS), joint range of motion, patient-reported functional scales (ASES, SANE, JOA) and MRI. By synthesizing these clinical data, the study aims to evaluate the extent of shoulder joint function restoration achieved by this technique, elucidate its potential advantages and limitations, and provide preliminary evidence-based data to inform future prospective comparative trials.

## 2. Methods

This study involved the collection of data from 44 patients who underwent arthroscopic surgery utilizing the “parachute” technique between January 2021 and January 2023 for the purpose of conducting a retrospective analysis. This study received approval from the Ethics Review Committee of Qingdao Central Hospital of Rehabilitation University (No. KY202506401). This study was exempted from the requirement to obtain informed consent from patients. The data were accessed for research purposes on 01/01/2026. All data were fully anonymized prior to access, authors don’t have access to information that could identify individual participants during or after data collection.

### 2.1. Patients

This retrospective study encompassed patients who underwent arthroscopic rotator cuff repair employing a modified Mason Allen “parachute” technique combined with BMS at our institution from January 2021 to January 2023. The inclusion criteria were as follows: (1) age between 35 and 80 years; (2) preoperative MRI confirming a full-thickness RCT; (3) surgical repair conducted using the “parachute” technique detailed below; and (4) a minimum follow-up duration of 24 months with comprehensive clinical and imaging data.

Systematically document the characteristics of the tears using data obtained from preoperative MRI and intraoperative arthroscopic examinations. All patients included in the study presented with full-thickness tears. Tear size was assessed preoperatively using MRI by measuring the maximum anteroposterior diameter of the torn tendon in the oblique coronal plane. In accordance with the Cofield classification: small (≤1 cm), medium (1–3 cm), large (3–5 cm), and massive (>5 cm). Within this cohort, tear size distribution was as follows: medium tears (2.0–2.5 cm) in 33 patients (27 patients with 2.0 cm tears and 6 patients with 2.5 cm tears), large tears (3.0–3.5 cm) in 7 patients (5 patients with 3.0 cm tears and 2 patients with 3.5 cm tears), and massive tears (5.0 cm) in 4 patients. No small tears (≤1 cm) were included in the study. The tear location is documented as involving either the isolated supraspinatus tendon or in combination with the infraspinatus tendon.

The exclusion criteria were as follows: (1) preoperative MRI indicating Goutallier grade 3 or higher fatty infiltration of the rotator cuff muscles; (2) a history of prior shoulder surgery on the affected side; (3) radiographic evidence of glenohumeral degenerative arthritis; (4) massive irreparable RCT characterized by severe retraction or advanced muscle atrophy; (5) concomitant shoulder pathologies potentially confounding functional outcomes, including shoulder instability, adhesive capsulitis, or fracture sequelae; and (6) severe medical comorbidities that contraindicated surgery or follow-up.

During this period, a total of 58 patients underwent surgical intervention utilizing the “parachute” technique. Among these, 11 patients were also diagnosed with shoulder arthritis, while 47 patients met the inclusion criteria for the study. However, three patients were lost to follow-up during the postoperative period, leading to incomplete follow-up data. Consequently, a total of 44 patients with complete follow-up data were ultimately included in this study.

The primary demographic information of the patients was collected, including variables such as gender and age. Preoperative and postoperative assessments were conducted with a minimum follow-up period of 24 months. These evaluations included the American Shoulder and Elbow Surgeons (ASES) score, the Single Assessment Numeric Evaluation (SANE), the visual analog scale (VAS) for pain, and measurements of active range of motion in forward flexion, external rotation, and internal rotation.

### 2.2. Methods

**Fig 1:**
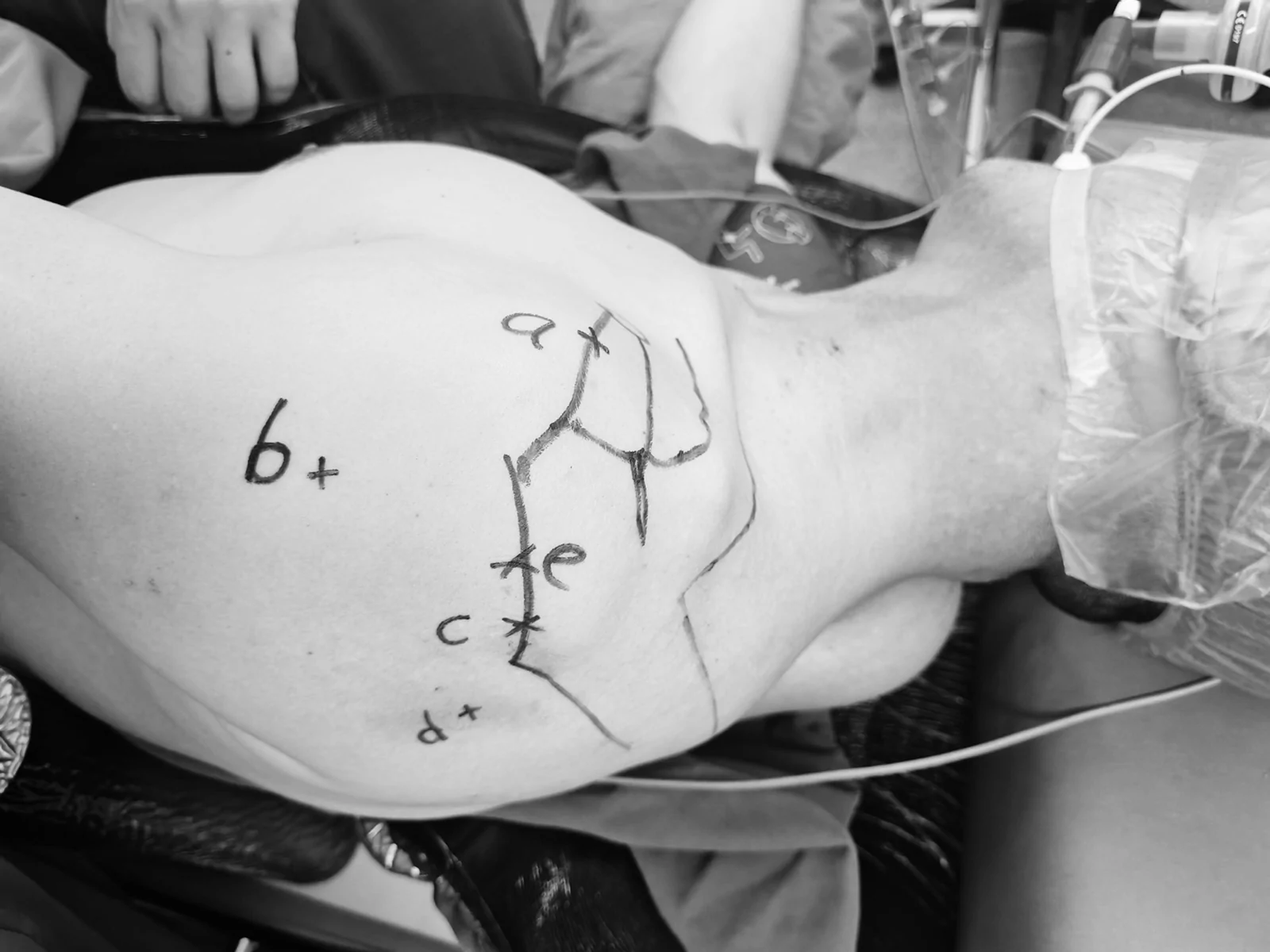
Preoperative marking of the surgical approach. (Fig 1 is an original work created by the authors’ research team and has not been previously published in any journal or other publication. There are no issues related to copyright transfer or licensing.).

The patient had lateral recumbent position under general anesthesia and cervical plexus block. The surgical technique employed five distinct approaches: the posterior-lateral approach, which is situated 2 cm inferior and 1 cm medial to the posterior-lateral corner of the acromion (Fig 1d); the anterior-lateral approach, located 2 cm inferior to the anterior corner of the acromion and lateral to the coracoid process (Fig 1a); the anterior-lateral approach, positioned 3 cm from the distal end of the acromion (Fig 1b); the posterior-lateral approach, which is found 2 cm anterior to the posterior-lateral corner of the acromion and 1 cm from the distal end of the acromion (Fig 1c); and the BMS channel and anchoring approach, located in the vicinity of the acromion near the midline (Fig 1e). Following the debridement of the subacromial synovial tissue, the posterior-lateral approach functioned as an observation channel, while the posterior and anterior approaches served as entry points for the suturing hook, and the anterior-lateral approach was utilized for manipulation.

Initially, a 30°arthroscope is employed to assess the shoulder joint. Should a pathological condition of the long head of the biceps tendon be identified, either tendon release or fixation is necessitated. In cases where the patient presents with preoperative adhesions, a capsula articularis release is indicated. During the examination, it is imperative to evaluate the extent of the RCT. The 30°arthroscope is subsequently reinserted through a posterior approach into the subacromial space, where a subacromial synovectomy and decompression are conducted via an anterolateral approach. In instances of observed subacromial impingement, acromioplasty should be performed either prior to or following the rotator cuff repair, contingent upon the condition of the subacromial space. Following this, the arthroscope is transitioned from the posterior approach to the posterolateral approach to assess the morphology and extent of the RCT. To identify the location of the outer edge of the cartilage of the humeral head and ascertain the number of medial anchor screws required (either one or two) based on the available area of the torn tendon footprint.

Under the guidance of a lumbar puncture needle, a BMS approach should be established, and anchor screws must be inserted through an approach located on the lateral aspect of the acromion, in proximity to the midline. Utilizing BMS technology, bone marrow outlets were created within the rotator cuff footprint area using a 2 mm diameter drill bit. Drilling was performed to a depth of 5 mm, with holes spaced 5 mm apart, extending from the edge of the articular cartilage to the lateral ridge of the greater tuberosity. Drilling continued until blood clots emerged from the bone marrow space, indicating adequate stimulation. The number of BMS holes was not fixed but determined by the size of the exposed footprint area. In the cases included in this study, the number of holes for each patient was tailored according to individual tear size and footprint dimensions. Based on the Cofield classification system, medium tears required 6–8 holes, large tears 8–10 holes, and massive tears 10–12 holes. This method ensures comprehensive biological augmentation of the entire footprint area while minimizing unnecessary bone damage. The first anchor screw should be positioned approximately 2 mm from the lateral edge of the joint, adjacent to the anterior margin of the RCT (posterior to the long head of the biceps tendon). The second anchor screw should be inserted 5–10 mm posterior to the first anchor screw.

**Fig 2:**
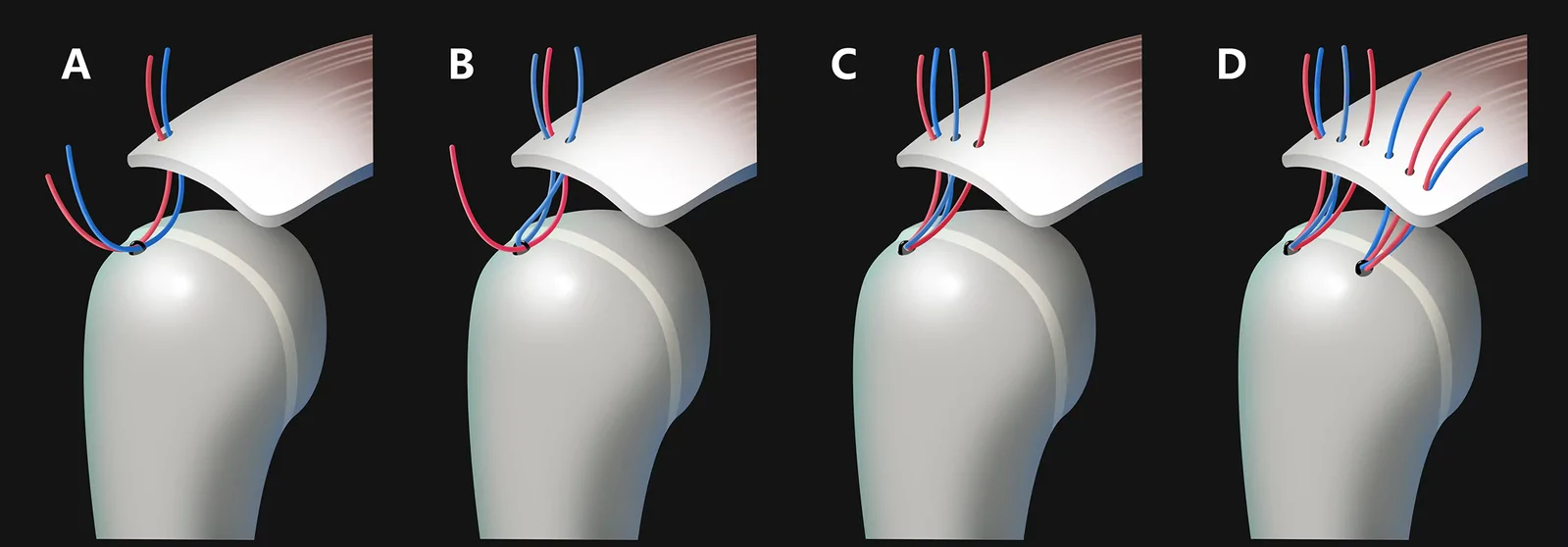
Threading steps for “parachute” structure.

**Fig 3:**
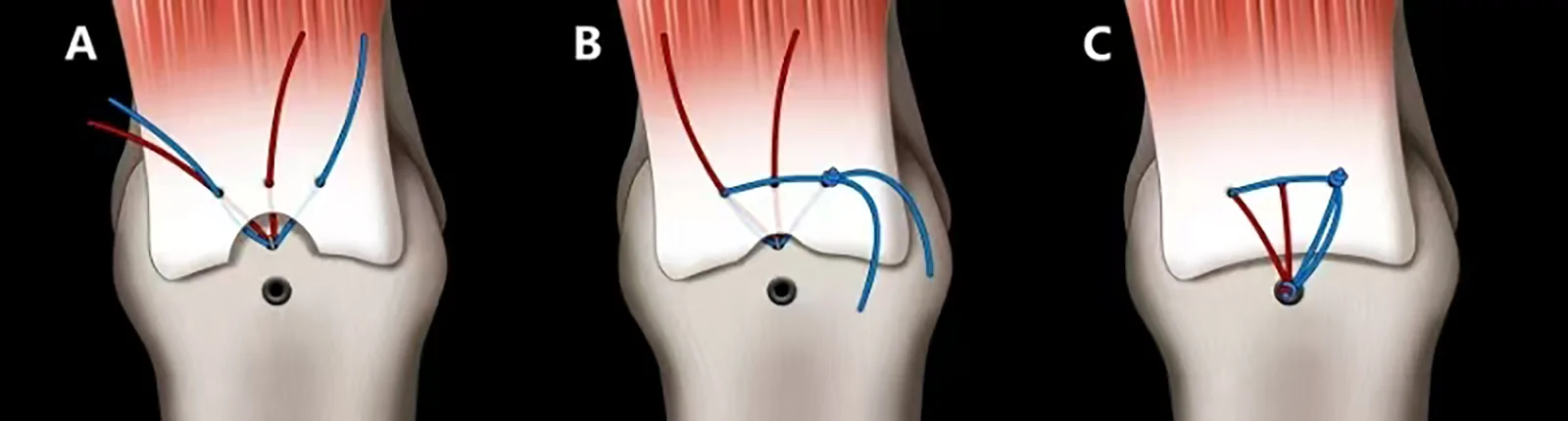
The method of small tear knotting and the final rendering of the “parachute” structure.

In the process of suturing the anterior segment of a torn rotator cuff, a banana-shaped suture hook (SutureLasso, Arthrex) is introduced into the subacromial space utilizing an anterior approach. Employing tissue forceps, the rotator cuff is gently elevated to ensure that the SutureLasso penetrates the full thickness of the tendon as vertically as feasible. The entry point should be positioned 10–12 mm medial to the outer edge of the RCT, as close to the inner side of the tendon as possible, in order to optimize the volume of lateral tendon available for pressurisation. The SutureLasso functions as a suture passer, capturing the tail of the non-absorbable suture and threading a size 1 polydioxanone suture (PDS, Ethicon) through it. During the initial threading process, both tails of the anchor (blue and red) are passed through the proximal edge of the tendon at the same entry point, with the exiting suture being retrieved through the lateral approach (Fig 2A). During the second pass, the SutureLasso was introduced into the rotator cuff at a location 1 centimeter distal to the initial puncture site, facilitating the passage of the third tail suture (blue) through the proximal tendon (Fig 2B). In the third pass, the SutureLasso again entered the rotator cuff at a point 1 centimeter distal to the second puncture site, allowing for the passage of the fourth tail suture (red) through the proximal tendon (Fig 2C). Patients with major tears develop symmetrical and identical structures (Fig 2D). The sutures that have been advanced to the joint surface are subsequently retrieved through a lateral approach. The tail suture introduced during the third pass is tied to the corresponding colored tail suture from the first pass (red) to create a modified Mason-Allen structure “Parachute” (Fig 3B,3C), which just comprises one Tennessee knot. At this juncture, the four tail sutures on the anchor have traversed the tendon three times (Fig 3A); specifically, two of the sutures have passed through the tendon once via the same aperture, while the remaining two have penetrated the tendon twice.

**Fig 4:**
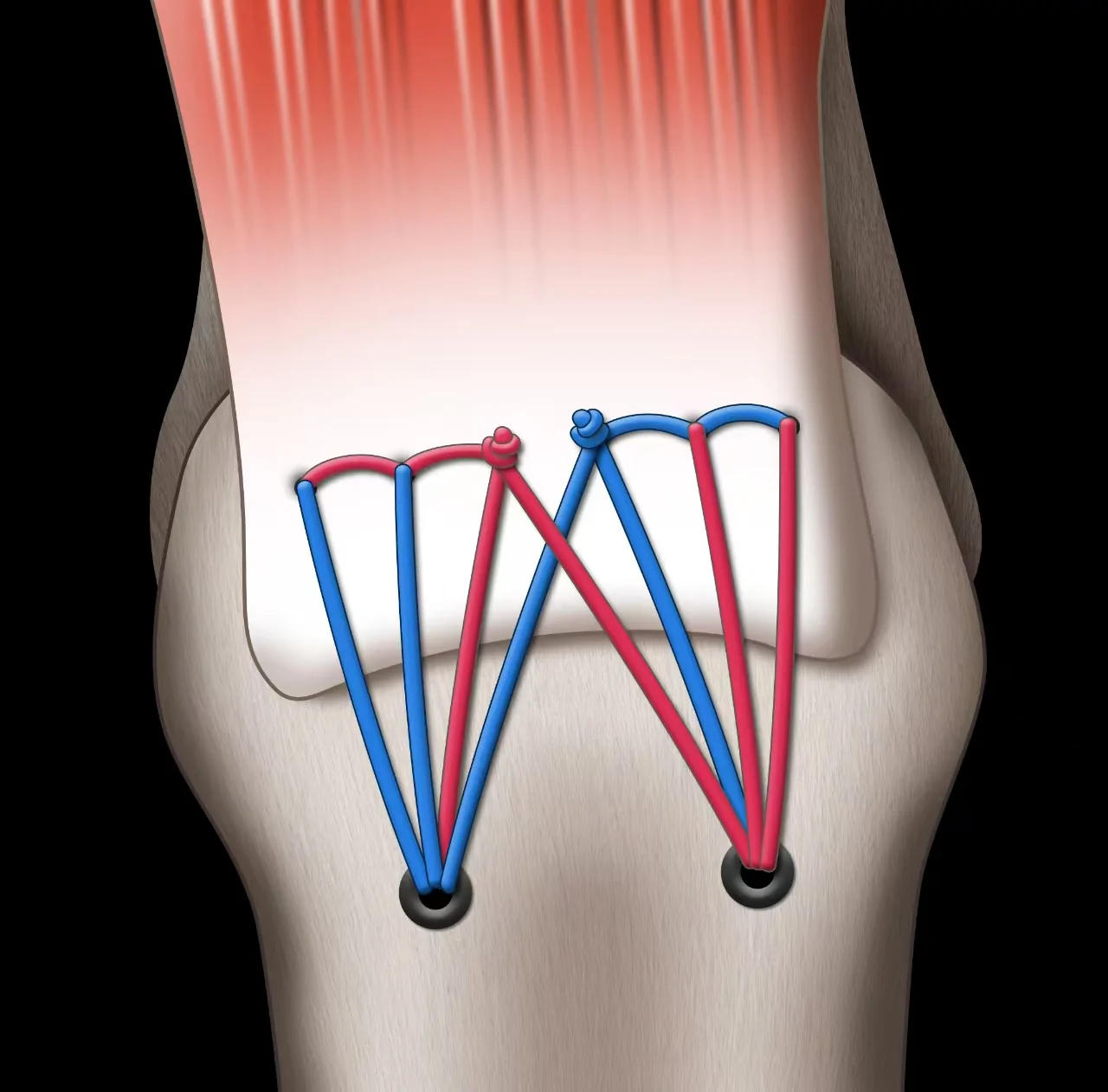
Final depiction of a substantial rotator cuff tear.

In cases where an increase in the number of anchor screws on the posterior inner side is necessitated by a substantial area of tearing, and when it is required to augment the number of “parachutes” utilized in the enhanced Mason-Allen “parachute” technique, it is advisable to store the sutures within the anterior superior approach channel to prevent entanglement. The technique for passing the tail line of the second anchor screw mirrors that of the first; it is particularly crucial that, during the passage through the tendon, both tail lines (designated as blue and red) from the second anchor screw are concurrently threaded through the same puncture hole located at the proximal edge of the tendon. The knots of the tail lines from both anchor screws should be strategically positioned in the central region of the torn tendon. It is imperative that all tail lines from the anchor screws are retained and not severed. Upon completion of the tying process, the tail lines should intersect and be secured with anterior and posterior external anchor screws, positioned 1 centimeter from the outer edge of the large knot footprint, thereby facilitating the construction of a suture bridge across the tendon surface. The arrangement of the anchor screws in the anterior-posterior orientation should be modified in accordance with the dimensions of the RCT, extending as far anteriorly and posteriorly as feasible to optimize the area of pressure contact (Fig 4).

All patients adhered to a standardized, stage-specific postoperative rehabilitation protocol. Phase 1 (0–6 weeks): The shoulder was immobilized using a sling equipped with a small abduction pillow for a duration of six weeks. Passive range of motion exercises, including forward flexion and external rotation within a pain-free range, were initiated on the first postoperative day. Patients were positioned in the supine position to minimize gravitational stress on the repair site. Active shoulder abduction and forward elevation were strictly contraindicated during this phase. Pendulum exercises were permitted beginning at two weeks postoperatively. Phase 2 (6–12 weeks): At six weeks post-intervention, active-assisted range of motion exercises were commenced, advancing to full active range of motion by twelve weeks. External rotation was restricted to 30° prior to eight weeks. Phase 3 (12 weeks and beyond) involved the initiation of strengthening exercises at 12 weeks, with a gradual progression to sport- and work-specific activities occurring after 4–6 months. Patients were advised to refrain from lifting heavy objects or engaging in overhead activities for a minimum of six months postoperatively. Compliance with these recommendations was assessed at each outpatient visit.

### 2.3. Methods of assessment

The Japanese Orthopaedic Association (JOA) score is a comprehensive assessment tool that ranges from 0 to 100 points. It includes several components: pain (30 points), functional capacity (20 points, encompassing general function and activities of daily living), range of motion (30 points, assessing active elevation, external rotation, and internal rotation), radiographic assessment (5 points), and joint stability (15 points). Higher scores correspond to better shoulder function. In patients undergoing rotator cuff repair, a JOA score of ≥83 points has been validated as the threshold for differentiating excellent or good outcomes from fair or poor outcomes. This scoring system was utilized for clinical evaluation and was measured at three distinct time points: prior to surgery, six months postoperatively, and at the final follow-up, which took place 24 months after the surgical intervention.

The evaluation of the range of motion for flexion and external rotation is performed using a protractor, while the measurement of internal rotation is established by identifying the highest spinal level at which the patient can place the back of their hand.

Before the surgical intervention, the dimensions of the RCT were assessed by measuring the distance from the lateral margin of the greater tuberosity to the edge of the supraspinatus tendon, using MRI. The evaluation of the repaired rotator cuff for retear was conducted two years post-surgery utilizing MRI. All MRI scans were independently evaluated by two radiologists who were blinded to the clinical outcomes. The Sugaya classification is presently the most widely utilized MRI-based system globally for evaluating the integrity of the rotator cuff tendon following repair surgery. This classification categorizes the postoperative status of rotator cuff repair into five distinct types. Type I demonstrates adequate tendon thickness with a uniform signal. Type II exhibits sufficient tendon thickness accompanied by localized areas of increased signal intensity. Type III is characterized by tendon thinning without disruption of continuity. Type IV presents with minor continuity disruptions, whereas Type V involves extensive continuity disruptions. Based on this classification, Sugaya types I, II, and III are typically regarded as indicative of healed or successfully repaired tendons, while types IV and V are classified as retears. In instances of disagreement between the two radiologists, consensus was achieved through deliberation.

### 2.4. Statistical analysis

All statistical analyses were performed using Origin Pro (OriginLab Corporation, Northampton, MA, USA). Quantitative data are presented as means with their corresponding standard deviations.

For comparisons between preoperative and 24-month postoperative measurements, paired-sample t-tests were conducted for the following variables: VAS, SANE, ASES, forward flexion, external rotation, and internal rotation. Due to each outcome measure involved only a single comparison between two time points, no adjustments for multiple comparisons were necessary.

A one-way repeated-measures analysis of variance (ANOVA) was conducted to evaluate differences in JOA scores across three time points: preoperative, 6 months postoperative, and 24 months postoperative. Upon identifying a significant overall effect by ANOVA, post-hoc pairwise comparisons were performed using the Bonferroni correction to control for multiple comparisons. The significance threshold for these comparisons was adjusted to α = 0.05/3 ≈ 0.017.

All statistical tests were two-tailed, and a p-value less than 0.05 was considered statistically significant unless otherwise specified.

## 3. Results

A total of 44 patients, comprising 18 males and 26 females, underwent the Modified Mason-Allen Suture Bridge “Parachute” Technique. The mean age of the participants was 62.0 ± 9.7 years. The mean tear size was 2.92 ± 1.21 cm, with a range of 2.0 to 5.0 cm. Based on the Cofield classification, 33 patients (75.0%) presented with medium tears (2.0–2.5 cm), 7 patients (15.9%) with large tears (3.0–3.5 cm), and 4 patients (9.1%) with massive tears (5.0 cm). Regarding tear location, 33 cases involving isolated supraspinatus tendon injuries and 11 cases involving combined injuries of the supraspinatus and infraspinatus tendons. Detailed information regarding the included patients, such as their age, tear type, tear size, and tear location, is provided in the S1 File. Besides, according to the preoperative MRI, the degree of fatty infiltration in the supraspinatus muscle was assessed using the Goutallier classification. As an exclusion criterion for this study was a Goutallier grade of 3 or higher, only patients with grades 0–2 were included in this group. The distribution of these grades is as follows: Grade 0 in 15 patients (34.1%), Grade 1 in 21 patients (47.7%), and Grade 2 in 8 patients (18.2%).

**Fig 5:**
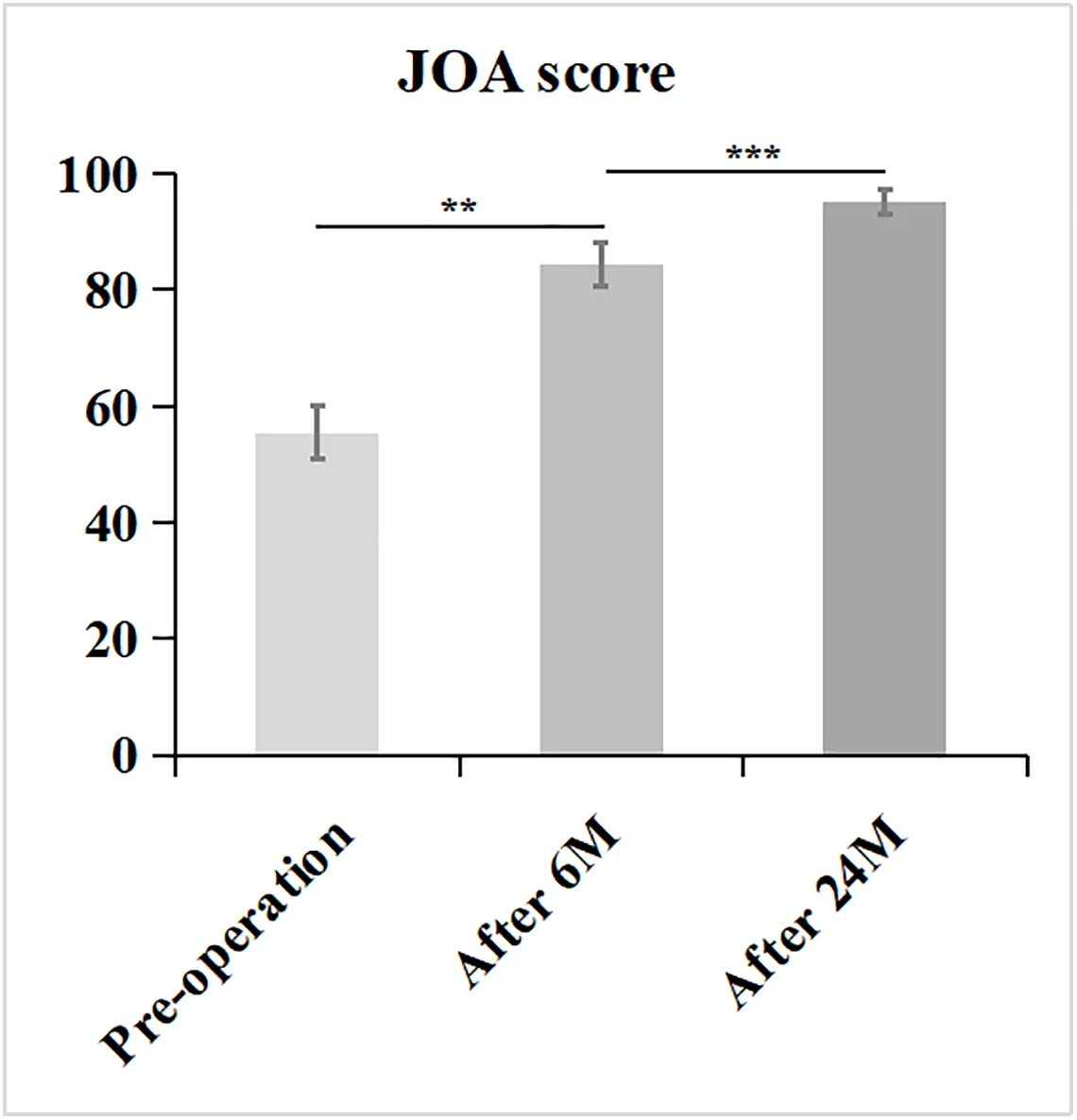
Follow-up assessments of JOA scores were conducted preoperatively, at 6 months and 24 months postoperatively. Data were analyzed using one-way repeated-measures ANOVA, followed by post-hoc pairwise comparisons with Bonferroni correction. Statistical significance was set at **P < 0.017 for pairwise comparisons (Bonferroni-corrected threshold) and ***P < 0.001 for the overall time effect.

**Fig 6:**
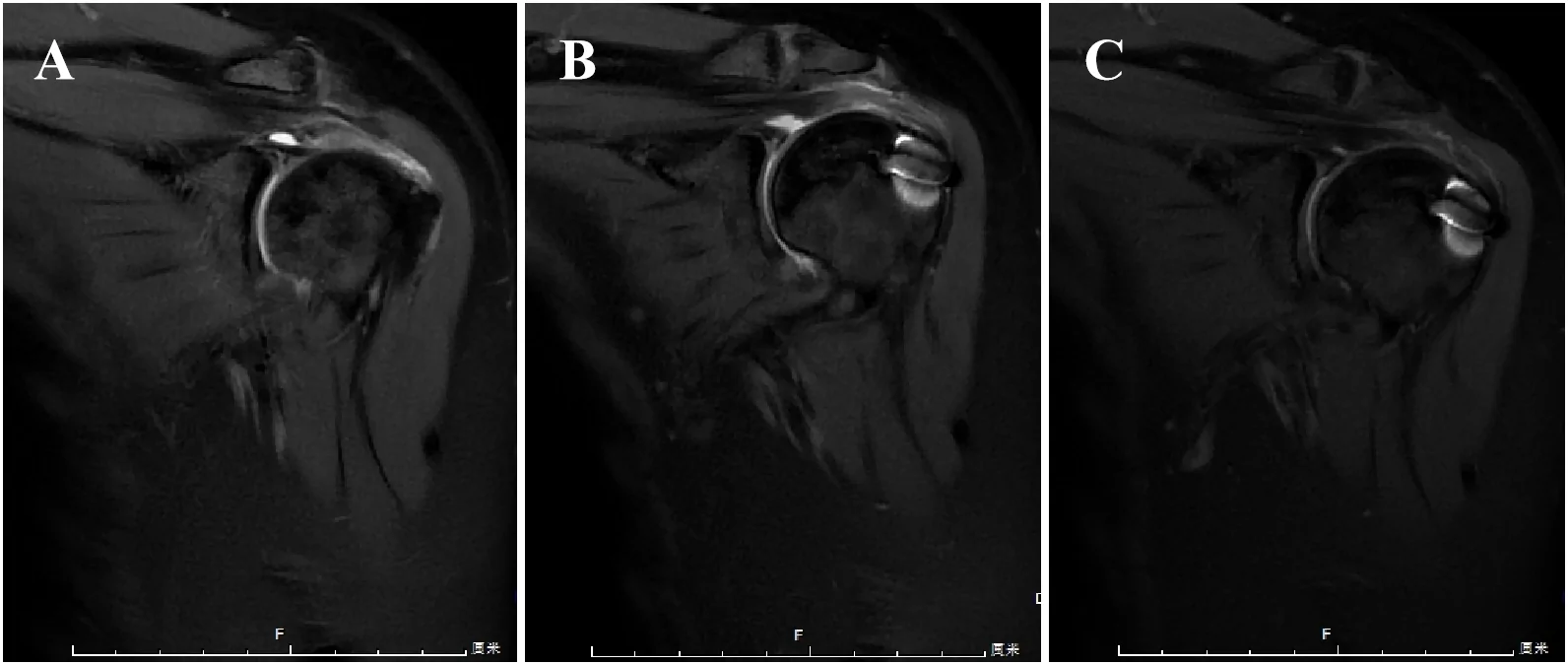
Follow-up assessments of MRI were conducted preoperatively (A), at 6 months (B) and 24 months (C) postoperatively.

According to the final follow-up evaluation, significant improvements were observed in VAS scores, SANE scores, ASES subjective shoulder scale scores and range of motion following surgery (Tables 1 and 2). Data are presented as mean ± standard deviation. Notably, JOA scores at both the 6-month and 24-month postoperative follow-ups continued to exhibit statistically significant differences (Fig 5). During the 24-month follow-up period, MRI evaluations, classified according to the Sugaya system, revealed the following distribution: 18 patients (40.9%) were categorized as type I, 19 patients (43.2%) as type II, and 7 patients (15.9%) as type III. No patients were classified as type IV or V. Consequently, all 44 patients (100%) demonstrated radiologically confirmed healing. Furthermore, no evidence of recurrence was observed at the final follow-up (Fig 6). Furthermore, no intraoperative or postoperative complications were reported. Specifically, there were no instances of nerve or vascular injury, wound infection, or complications related to suture anchors among the patients.

**Table 1:** Comparison between preoperative and postoperative clinical outcomes. *Statistically significant (P < 0.05).

	Pre-operative	Post-operative	*P* value
**ASES**	**42.70 ± 8.34**	**89.23 ± 6.66**	<0.001*
**SANE**	**53.82 ± 8.99**	**92.18 ± 5.55**	<0.001*
**VAS**	**5.48 ± 1.42**	**1.02 ± 0.73**	<0.001*

**Table 2:** Comparison between preoperative and postoperative active range of motion. FF means forward flexion, ER means external rotation, IR means internal rotation. *Statistically significant (P < 0.05).

	Pre-operative	Post-operative	*P* value
**FF**	**128.09 ± 22.70°**	**160.64 ± 14.59°**	<0.001*
**ER**	**47.39 ± 11.33°**	**59.27 ± 8.95°**	<0.001*
**IR**	**T10.89 ± 1.47**	**T8.43 ± 1.35**	<0.001*

## 4. Discussion

RCT represent a prevalent shoulder condition within the elderly demographic [33]. Despite the favorable clinical outcomes associated with arthroscopic rotator cuff repair, the attainment of tendon healing continues to encounter numerous challenges [21,34,35]. The healing process of tendons is primarily influenced by two key factors: the strength of mechanical fixation and the degree of biological healing. An enhanced Mason-Allen suture technique was employed, integrating the “Parachute” method with BMS for the repair of rotator cuff injuries. The objective is to establish a dual approach that improves both biomechanical performance and biological healing outcomes. This study retrospectively assessed the functional outcomes of 44 patients with RCT who underwent the modified Mason-Allen procedure at our institution between January 2021 and January 2023. The findings demonstrated a significant improvement in the patient-reported ASES score, which increased from 42.70 ± 8.34 preoperatively to 89.23 ± 6.66 postoperatively (P < 0.001). Similarly, the SANE score rose from 53.82 ± 8.99 to 92.18 ± 5.55 (P < 0.001). The VAS score for pain significantly decreased from 5.48 ± 1.42 prior to surgery to 1.02 ± 0.43 at the final follow-up (P < 0.001). Based on the clinical and imaging follow-up data presented, this study demonstrates that the modified Mason-Allen “parachute” technique combined with BMS is both feasible and safe for the treatment of RCT. Postoperative functional scores and joint range of motion were significantly improved, and no serious complications were observed during the mid- to long-term follow-up period. It is important to note that, as a retrospective case series, this study design only allows for the demonstration of the technique’s effectiveness and operability within this specific patient population and does not permit conclusions regarding its superiority over other surgical methods. These findings provide preliminary clinical evidence to support further investigation through prospective, randomized controlled trials.

The enhanced Mason-Allen suture technique signifies a notable advancement in the mechanical strength associated with the repair of RCT and has gained widespread acceptance in clinical practice [36,37]. This technique optimizes both the configuration of the suture and its biomechanical properties, thereby substantially improving the reliability of the repair. The primary innovation resides in the simplification of the conventional rectangular suture structure into a composite arrangement that incorporates horizontal mattress sutures alongside vertical simple sutures. This design effectively addresses the principal limitations of traditional techniques while preserving a majority of the tensile strength. Despite the reduced knot volume associated with the improved Mason-Allen suture technique in comparison to traditional methods, there remains a risk of impingement in patients exhibiting a narrowed acromiohumeral interval. Although the sliding locking knot technique is effective in compressing the knot, excessive tension may result in suture creep, leading to a loss of tension. From a technical design standpoint, the proposed “parachute” suture configuration exhibits distinctive mechanical distribution properties. This design theoretically facilitates multidirectional tension distribution and uniform compression across the tendon surface by combining the firm grip of the inner row of anchors with the bridge-like compression exerted by the outer row of anchors. Such a configuration may biomechanically enhance the tendon-to-bone contact area and mitigate local micromotion. Nevertheless, it is crucial to underscore that, as this study did not include direct biomechanical testing or animal experiments, these assertions regarding mechanical optimization remain theoretical and necessitate validation through future fundamental research.

Previous studies have extensively demonstrated the biological efficacy of BMS technology in promoting tendon healing by recruiting bone marrow mesenchymal stem cells and releasing growth factors [21,32,33]. In the present study, we sought to modify the knot position to be closer to the BMS drilling site. The underlying rationale for this modification was to establish a relatively enclosed microenvironment locally, facilitated by the coverage effect of a “parachute” structure, with the aim of minimizing the loss of bone marrow components into the joint cavity. Nonetheless, it is important to acknowledge that this hypothesis was not directly tested in the current study and therefore should not be regarded as a definitive conclusion. Future investigations, including animal experiments or prospective studies employing histological analysis or biomarker detection, are planned to specifically validate this hypothesis. By employing stem cell recruitment, growth factor release, and angiogenesis, we significantly enhance the functional integration of the tendon-bone interface, thus expediting the overall treatment process. Our results indicate that all patients experienced significant improvements in clinical scores and joint mobility, accompanied by a notable enhancement in quality of life.

This study possesses inherent limitations characteristic of retrospective observational research. Primarily, the absence of a control group constitutes the most significant methodological limitation of this study. As a single-arm retrospective case series, it is unable to establish a causal relationship between the observed functional improvements and any specific component of the intervention. The modified Mason-Allen “parachute” suture configuration, the BMS procedure, or their combined effect. It remains plausible that factors such as patient selection bias, the natural progression of the disease, or confounding variables, including tear size, tendon quality as indicated by the Goutallier grade, patient age, comorbidities, and adherence to postoperative rehabilitation. These reasons may have influenced the favorable outcomes. Therefore, the primary conclusions should be interpreted with caution. These findings are presented as proof of concept and clinical feasibility rather than as evidence of superiority or definitive treatment efficacy. Secondly, the data were derived from a single center with a limited sample size. Although we have thoroughly documented the characteristics of rotator cuff injuries in each case, the relatively small sample size precludes meaningful subgroup analyses to identify which patient groups may derive the greatest benefit from this technique. Variations in tear size, tendon quality, and rehabilitation adherence among patients from different institutions necessitate future multicenter studies with larger cohorts for validation. Thirdly, the mechanistic inferences discussed concerning the “parachute” structure’s role in enhancing mechanical distribution and reducing bone marrow seepage in the BMS area are primarily grounded in theoretical design principles and prior literature. These inferences were not directly substantiated by histological, biomechanical, or synovial fluid composition analyses within the present study. Consequently, these points should be interpreted as conceptual explanations of the technical design and as suggestions for future research directions, rather than definitive causal explanations for the current findings.

Building upon the findings of this retrospective analysis, we intend to pursue the following initiatives. First of all, design a prospective, randomized, controlled trial to directly compare the modified and standard Mason-Allen techniques, with primary endpoints including healing rates confirmed by MRI at one year postoperatively and functional outcome measures. Secondly, assess the applicability of the modified technique across diverse tear patterns and develop individualized suture protocols. Thirdly, conduct biomechanical investigations to quantify the maximum failure load of the modified technique. Despite these limitations, the present study offers valuable insights for clinical practice. The modified Mason-Allen technique implemented at our center is feasible and demonstrates satisfactory functional recovery of the shoulder joint during follow-up.

## 5. Conclusion

This retrospective analysis indicates that the modified Mason-Allen “parachute” technique for the treatment of RCT can result in favorable postoperative functional recovery, substantial pain reduction, and marked enhancement of joint mobility. Nevertheless, given the inherent limitations of the study design, these findings require validation through prospective randomized controlled trials. In future clinical practice, the selection of the surgical technique will be tailored to the specific tear type in each patient, with an emphasis on the implementation of standardized rehabilitation protocols.

## Supporting information

S1 File Case information and scoring data. The dataset includes information on patients’ age, tear type, tear size, tear location, Goutallier grade, as well as their scores and range of motion measurements.
(ZIP)
