# Supplementary material for: Retrospective analysis of arthroscopic repair outcomes using a modified Mason-Allen suture bridge “parachute” technique with bone marrow stimulation for rotator cuff tears
Source: PLoS One. 2026 Aug 20;21(8):e0356245. doi: 10.1371/journal.pone.0356245 (PMC13492772; doi:10.1371/journal.pone.0356245)
Supplement: Supplementary file 1 [file pone.0356245.s003.docx]

Reviewer #1: This paper discusses the management of rotator cuff tears with a unique surgical technique. This surgical technique combines a modified Mason Allen suture bridge with bone marrow stimulation. The topic is clinically relevant, and the authors report good clinical outcomes and structural healing at an extended follow up period of 24 months. However, in its current form the manuscript has major methodological and reporting limitations that need to be addressed.
It is unclear what patient population this study has been conducted in as the specific tear (i.e. supraspinatus), tear size or tear type (partial or full thickness) has not been reported. Furthermore, the paper reports that the presence of rotator cuff lesions was an exclusion criteria. It is unclear what these lesions refer to. Furthermore, the authors should clearly report on tendon quality given Goutallier grade 3 or higher fatty infiltration was excluded. Concomitant procedures should also be reported alongside number of anchors used. Rehabilitation protocols should also be reported.

Response:

We sincerely appreciate your thorough and detailed comments, which have been instrumental in enhancing the manuscript's comprehensiveness and reproducibility. Each point has been addressed meticulously.

First, concerning the tear characteristics of the patient cohort, the revised manuscript has been supplemented with a comprehensive description of the tear features. All 44 patients in this group presented with full-thickness rotator cuff tears, classified according to the Cofield system as medium-sized in 33 cases (75.0%), large in 7 cases (15.9%), and massive in 4 cases (9.1%). In all patients, the supraspinatus tendon was involved, with some tears extending to the infraspinatus tendon.

Second, regarding the clarification of the exclusion criteria for "rotator cuff lesions," the original statement "excluding rotator cuff lesions" was inaccurate and logically inconsistent with the study population, which comprised patients with rotator cuff tears. The intended exclusion criteria were other lesions that could confound shoulder joint function, such as shoulder instability, frozen shoulder, or sequelae of fractures. This has been rectified in the revised manuscript, where these specific conditions are explicitly enumerated.

Third, concerning the reporting of tendon quality, since patients with a Goutallier grade of 3 or higher were excluded, it is essential to provide the distribution of Goutallier grades among the enrolled patients. This information has been incorporated into the baseline characteristics table in the revised manuscript.

Fourth, concerning the number of anchors utilized, the revised manuscript has been supplemented with detailed information regarding the anchor configuration.

Fifth, regarding the rehabilitation protocol, we have elaborated the description in the Methods section by transforming the initial brief overview into a detailed, staged standardized rehabilitation protocol. This includes specifications of activity restrictions, allowed movements, and progression schedules at each stage, thereby enhancing its utility as a reference for other clinical centers.

All the aforementioned modifications have been highlighted in yellow in the revised manuscript. We believe that these additions have enhanced the completeness, clarity, and reproducibility of the reporting of the study population characteristics in accordance with academic standards.

Minor comments:
The manuscript would benefit from substantial English language editing. There are frequent grammatical issues, awkward phrasing, and terminology inconsistencies that reduce readability.
The reference list contains duplicated references including for example 4 and 9. 10 and 11.

Response:

We sincerely appreciate your valuable suggestions for improvement and fully acknowledge your criticisms. The original manuscript contained issues related to grammar, awkward phrasing, and inconsistent terminology, which adversely affected its overall readability. Furthermore, the references necessitated additional verification and refinement. We have systematically addressed both of these concerns.

Following this revision, we engaged a professional peer with expertise in editing English for medical journals to perform a final review of the manuscript, ensuring that the language adheres to publication standards. Additionally, we meticulously examined the reference list, identified and rectified duplicate citations, and reordered all citation numbers to guarantee precise correspondence between in-text citations and the reference list. We sincerely appreciate your careful attention to the details of the references.

Introduction:
The introduction is straightforward. The manuscript would benefit from discussing retear rates for bone marrow stimulation in cuff repairs. Second, the introduction blends technical description with anticipated benefits in a way that appears promotional rather than scientific. Given this is a retrospective case serious, the introduction should frame this as an evaluation of the technique in practice.

Response:

We sincerely appreciate the reviewers’ valuable comments. We concur that a comprehensive discussion regarding the impact of bone marrow stimulation (BMS) techniques on retear rates in the introduction is essential to substantiate the biological rationale underlying the combined technique employed in this study. In the original manuscript, BMS was only briefly mentioned, and quantitative clinical evidence demonstrating its effect on reducing retear rates was not adequately provided, which represents a notable limitation. We also acknowledge and accept the reviewers’ critique concerning the use of promotional language in the introduction, which is inappropriate for a retrospective observational study. The primary objective of this study is not to advocate for the superiority of a novel technique but rather to objectively assess its performance in terms of safety, feasibility, and early functional recovery based on real-world clinical data, as well as to identify its potential advantages and limitations.

To address these concerns, we have considerably expanded and enhanced the introduction by incorporating recent high-quality systematic reviews and meta-analyses, with explicit citations of data from other researchers. This body of evidence robustly supports that the combined application of BMS in rotator cuff repair can significantly reduce postoperative retear rates, thereby establishing a strong evidence-based medical rationale for selecting this technique. Furthermore, in response to your recommendations, we have revised the concluding paragraph of the introduction by eliminating speculative and promotional language and adopting a more neutral and objective scientific tone to accurately convey the original intent of this retrospective study design.

We sincerely thank the reviewers for their thorough evaluation of this manuscript once again. All the aforementioned revisions have been incorporated into the revised version and are clearly highlighted. We believe that these modifications have comprehensively addressed the reviewers' comments and have substantially improved the scientific rigor and accuracy of the paper. Should there be any further suggestions, we remain willing to make additional improvements.

Methods:
The methods section requires major revision. Inclusion and exclusion criteria require clarification. Specifically, tear characteristics need to be clearly recorded. This includes full thickness vs partial thickness tears, tear size, tear location (in which rotator cuff tendon). Furthermore, the methods describes exclusion of patients with rotator cuff lesions which is inconsistent given this is a population with rotator cuff tears.
It is unclear how it was determined that repairs were intact or ‘healing’. Please report using a standardised classification such as Sugaya.
Statistical analysis methods were not reported on. It is unclear what statistical tests were used to determine significant results. This must be reported.

Response:

We sincerely appreciate the reviewer's valuable suggestions. We will address each of your comments systematically and in detail.

1. Inclusion and exclusion criteria

We sincerely appreciate your insightful critique and detailed guidance concerning the methodology section. We fully acknowledge the validity of your comments. The original manuscript’s methodology section exhibited significant deficiencies: firstly, the inclusion criteria did not adequately specify tear characteristics; secondly, there was a logical inconsistency regarding the exclusion of patients with rotator cuff lesions. We will address each point individually and outline the corresponding revisions implemented.

First, the study population consisted of patients with rotator cuff tears; however, the exclusion of "rotator cuff lesions" presents a logical inconsistency. This issue arose from a translation and expression error. The original intent was to exclude other rotator cuff-related pathologies that could confound the outcomes, aside from the rotator cuff tear itself. Nevertheless, the term "rotator cuff lesions" is imprecise and may be easily mistaken for "rotator cuff tears." In the revised version, this contradictory statement has been removed, and the inclusion and exclusion criteria have been reorganized to reflect a clear and logical hierarchy.

Second, concerning the type of tear, a thorough review of the original medical records and intraoperative notes for all 44 patients in our cohort confirmed that each presented with full-thickness rotator cuff tears; no cases of partial tears were included. This criterion was explicitly specified in the inclusion criteria.

Third, with respect to tear size, the measurement methods and classification standards have been clearly documented in the revised manuscript.

Fourth, concerning the location of the tears, we have enhanced the description of their anatomical sites in the revised manuscript. Specifically, 33 cases involved isolated supraspinatus tendon tears, while 11 cases involved combined tears of the supraspinatus and infraspinatus tendons. Corresponding baseline tables have also been incorporated into the Results section.

In the revised manuscript, we have systematically revised the inclusion and exclusion criteria presented in Section 2.1 of the Methods. Concurrently, we have updated the baseline description in the Results section. We believe that these comprehensive revisions adequately address your concerns, resulting in a description within the Methods section that is clear, complete, and logically coherent.

2. Criteria for Healing

We sincerely thank the reviewers for their rigorous and precise comments. We fully agree that the original manuscript's assessment of tendon healing status lacked an objective and standardized classification system, and that using only a general description of "complete or partial healing" is insufficiently standardized in the field of rotator cuff repair research.

In response to this issue, we have re-evaluated and reported all postoperative 24-month MRI images using the Sugaya classification system in the revised manuscript. The Sugaya classification is currently the most widely adopted MRI evaluation system internationally for assessing postoperative rotator cuff repair, categorizing repair status into five types. Type I indicates sufficient rotator cuff thickness with a uniform signal; Type II denotes sufficient thickness with a partial high signal; Type III reflects thinning of the rotator cuff without discontinuity; Type IV corresponds to a small tear in the rotator cuff; and Type V signifies a large tear in the rotator cuff. Types I through III are considered indicative of successful healing, whereas Types IV and V are classified as re-tears.

Two independent radiologists were invited to re-evaluate the postoperative MRIs of all 44 patients using a double-blind methodology. Among these cases, 18 (40.9%) were classified as Type I, 19 (43.2%) as Type II, and 7 (15.9%) as Type III; no cases were identified as Type IV or Type V. Consequently, all 44 patients demonstrated Sugaya Type I–III healing, with no imaging evidence of re-tear. These findings are consistent with the original manuscript’s assertion that "all patients healed." Notably, the current reporting approach employs standardized criteria that are quantifiable, comparable, and reproducible.

We have comprehensively expanded the criteria and evaluation procedures of the Sugaya classification in Section 2.3 of the Methods in the revised manuscript. Additionally, in the Results section, we substituted the prior general descriptions with detailed classification data and made corresponding modifications in the Abstract. All changes have been clearly highlighted. We are confident that these revisions adequately address your concerns and align the assessment of tendon healing with the standardized requirements of the field.

3. For Statistical analysis.

We sincerely appreciate your identification of the deficiencies in our description of the statistical methods. We concur that a detailed and precise explanation of statistical procedures is essential to ensure the reproducibility of research findings. In the original manuscript, the description of the specific statistical analyses was insufficiently detailed and lacked clear specification of the exact tests applied to each variable, as well as the correction methods employed for multiple comparisons. In response to your valuable feedback, we have extensively revised and expanded the statistical methods section in the updated manuscript.

The primary analysis of this study entailed comparing clinical indicators obtained before surgery with those collected at a 24-month postoperative follow-up. Given that all measurement indicators constituted paired data from the same cohort of patients at two distinct time points, paired-sample t-tests were employed for statistical evaluation. Specifically, paired comparisons were conducted for the following variables: clinical scores (VAS, SANE and ASES), joint range of motion (FF, ER and IR), and JOA scores, which were measured at three time points preoperatively, 6 months postoperatively, and 24 months postoperatively. For the analysis of JOA scores across these three time points, repeated-measures ANOVA was utilized for overall comparison, followed by pairwise post hoc tests.

Concerning the issue of multiple comparison corrections, we addressed two distinct scenarios separately. For the primary clinical indicators (VAS, SANE, ASES, FF, ER and IR), paired comparisons were conducted solely at a single time point (preoperative versus 24 months postoperative). Consequently, each indicator was tested only once, rendering multiple comparison correction unnecessary 4. In contrast, for the JOA score, which was measured repeatedly at three time points, the Bonferroni correction was applied during post-hoc pairwise comparisons to adjust the significance level and control the cumulative risk of Type I error.

We have incorporated all the aforementioned details into the revised statistical methods section and have accordingly updated the figure legends related to multiple comparisons of JOA. All modifications have been clearly highlighted. We believe that these revisions comprehensively address your concerns and enhance the description of the statistical methods to satisfy reproducibility standards.

Results:
The results section does not include key patient characteristics as outlined in comments above. Furthermore Table 2 is incorrectly labelled. The values suggest it is range of motion though it is labelled ASES, SANE and VAS.

Response:

We sincerely thank you for valuable feedback. We acknowledge that the original manuscript's results section included only basic demographic information, such as age and gender, and omitted critical baseline lesion characteristics, including tear type, tear size, and tear location. This omission represents a significant gap in the reporting of results. We have thoroughly addressed this issue in the current revision. Initially, we reported the tear characteristics of all 44 patients in the results section, detailing tear type, size, and location. Subsequently, we included a summary table of baseline characteristics in the supplementary materials to offer readers a clear and concise overview of lesion severity within the study population.

During the preparation of the tables, the column header format from Table 1 was inadvertently duplicated in Table 2. Consequently, the column headers in Table 2 incorrectly displayed the names of clinical scoring scales (ASES, SANE, VAS), whereas the data actually represented measurements of joint range of motion (FF, ER, IR). This formatting error was an oversight and has been rectified in the current revision. Additionally, we have thoroughly reviewed Table 1 to confirm the accuracy of its title, column headers, and data. We apologize for any inconvenience this formatting error may have caused the reviewers and assure that, following this revision, all table titles and contents are fully consistent and accurate.

Discussion:
The discussion currently significantly overstates the implications of the data and makes mechanistic claims. Whilst it is biologically plausible that the parachute structure can enhance sealing and bone marrow leakage, it is unclear whether this is true as it is not within the scope of the study and not mechanistically proven. Furthermore, reported mechanical benefits of the parachute technique can be suggested but they remain unproven and not within the scope of the results of this paper. The manuscript should suggest that this repair method supports feasibility rather than superiority compared to other techniques.

Response:

We sincerely appreciate your rigorous and valuable academic feedback. We acknowledge that the original manuscript contained instances of over-interpretation in the discussion section, extending beyond the data presented. Specifically, the theoretical assertion that the "parachute structure may reduce bone marrow leakage" was directly associated with clinical efficacy, and conclusions regarding mechanical performance were drawn without experimental validation. Such statements lack sufficient rigor for a retrospective study primarily centered on clinical functional evaluation. We fully accept your critique and have comprehensively revised and restructured the discussion section in response.

The fundamental principles guiding this revision encompass several key aspects. First, the analysis was refocused on the core data, restricting the discussion exclusively to the observed outcomes of this study, specifically improvements in clinical functional scores and radiological healing. Speculative conclusions unsupported by mechanical testing or comparative experiments were omitted. Second, absolute claims lacking direct empirical evidence, such as "enhanced sealing to reduce bone marrow leakage," were rephrased as "theoretically designed potential advantages" and identified as "research directions warranting future validation." Furthermore, throughout the manuscript, comparative statements such as "superior to traditional techniques" were consistently replaced with more precise descriptions centered on the study’s findings, including terms like "safe and feasible," "demonstrating favorable early clinical outcomes," and "offering an alternative treatment strategy."

We sincerely appreciate your thorough guidance and valuable suggestions. In response, we have substantially revised the discussion section and have clearly indicated the modifications made.

Reviewer #2: Reviewer Comments
I would like to thank the Editor for the opportunity to review this manuscript. The topic is clinically relevant, and the authors present an interesting surgical approach. However, I have several major concerns regarding the study design and reporting.
The most important limitation is the absence of a control group. Because the intervention combines a modified Mason-Allen “Parachute” technique and bone marrow stimulation (BMS), it is not possible to determine which component contributes to the reported outcomes. In addition, key information regarding patient characteristics and tear morphology is insufficient, and there are concerns about data presentation (e.g., Table 2).
Overall, the current manuscript does not provide sufficient evidence to support the authors’ conclusions.

Major comments
1. Lack of a control group
The most critical limitation is the absence of a control or comparison group. Because the intervention combines a modified Mason-Allen “Parachute” technique and BMS, it is unclear whether the favorable outcomes are attributable to the repair configuration, BMS, or patient selection. Therefore, the main conclusion is not sufficiently supported.

Response:

We fully acknowledge and concur with your principal criticism regarding the absence of a concurrent control group, which constitutes the most significant methodological limitation of this study. We have no objections to this point. In light of this candid acknowledgment, we wish to provide you with a brief explanation of the objective reasons for the exclusion of a control group, thereby clarifying the true context of the research.

Firstly, this study constitutes an exploratory retrospective analysis conducted during the initial clinical implementation of a novel surgical technique. The "parachute" technique was first introduced at our institution in 2021. The primary objective of the study was to assess the safety, feasibility, and early functional recovery associated with this new technique, rather than to establish its superiority over existing surgical methods. This approach aligns with the prevailing research paradigm for the initial evaluation of innovative surgical procedures.

Secondly, there was ethical and practical resistance encountered in clinical practice. Throughout the study, preliminary analyses indicated that both surgeons and patients showed a preference for the new technique. Since both groups recognized the potential benefits of the new method, randomly assigning patients to the traditional procedure group encountered substantial resistance during clinical implementation, a factor beyond the unilateral control of the researchers.

Thirdly, it is important to consider the learning curve and technical uniformity associated with new surgical techniques, particularly those involving specialized suture configurations and BMS procedures. These techniques exhibit a distinct learning curve during their initial implementation. To maximize consistency in surgical procedures and minimize technical variability throughout the study period, all consecutive cases meeting the inclusion criteria were incorporated into the observation group, with an emphasis on standardizing technical processes and accumulating data. Introducing a control group concurrently at this stage could have introduced significant confounding bias due to variability in surgeons' skill levels, which would be more pronounced than in the absence of a control group.

Notwithstanding the practical considerations previously mentioned, we acknowledge that explanations grounded solely in objective conditions cannot substitute for the necessary methodological rigor. Consequently, we have undertaken systematic revisions to the manuscript. In the limitations section, we have provided a detailed discussion emphasizing the objective reality that the observed effects cannot be ascribed to any particular intervention component. Throughout the manuscript, causal inference language has been consistently moderated to reflect descriptive associations. Furthermore, the conclusion explicitly advocates for future prospective randomized controlled trials. All these amendments have been incorporated into the revised manuscript and are clearly highlighted within the text.

We sincerely appreciate your thorough attention to this fundamental issue. It is through such rigorous methodological examination that the manuscript is able to achieve a higher academic standard following revision.

2. Insufficient patient and tear characterization
Line101-111
Key baseline information is missing, including:
• partial vs full-thickness tears,
• involved tendons (e.g., supraspinatus/infraspinatus/subscapularis),
• tear size and classification,
• Detailed Goutallier grade distribution.
Response:

Without these details, the reported healing rate cannot be appropriately interpreted.

We sincerely appreciate your emphasis on this critical methodological requirement. We concur that comprehensive baseline information is essential for the accurate interpretation of healing rates. In the original manuscript, we reported only basic demographic data, such as age and gender, without including detailed information on tear types, involved tendons, tear size classification, and Goutallier grading distribution. This omission limited readers’ ability to thoroughly evaluate the characteristics of the study population and the generalizability of the findings. In response to your valuable comments, we have systematically supplemented the manuscript to address these aspects.

3. Unclear definitions (healing and retear)
Line34
The definitions of “complete healing,” “partial healing,” and “retear” are not provided. Clear, reproducible criteria are required.

Response:

We sincerely appreciate the reviewers' thorough and insightful comments. We concur that the original phrase "complete or partial healing in all patients" in line 34 of the manuscript was overly vague and lacked a clear, standardized, and reproducible definition. To rectify this, we have implemented two substantive revisions.

In the Methods section 2.3, the corresponding Sugaya classification criteria for each term were explicitly defined. This classification system is widely used internationally and demonstrates strong inter-observer reliability, thereby offering a clear and reproducible framework for assessing healing.

Second, in the Abstract Results section, the ambiguous phrase "complete or partial healing" was replaced with precise outcomes based on the Sugaya classification. Following this revision, qualitative descriptors such as "complete" or "partial healing" were eliminated, thereby effectively addressing the issue of ambiguous terminology. All data were derived from consistent assessments conducted by two independent radiologists employing a double-blind methodology.

Third, in the Results section, we replaced the original vague descriptions with data based on the Sugaya classification.

All related changes have been highlighted in yellow in the revised manuscript. We believe that these modifications have rendered the healing assessment criteria clearer, more objective, and reproducible.

4. Inadequate description of BMS procedure
Line144-147
The number of perforations for BMS is not reported. More detailed and quantitative description is necessary for reproducibility.

Response:

We express our sincere gratitude to the reviewers for highlighting the insufficient detail in the description of the bone marrow stimulation (BMS) procedure. We concur that, as the central biological enhancement step in this technique, a comprehensive account of the operational parameters of BMS is essential to ensure the reproducibility of the study. While the original manuscript specified the drill bit diameter (2 mm), hole depth (5 mm), and hole spacing (5 mm), it omitted the precise number of perforations, which is a critical detail for enabling other surgeons to accurately replicate the procedure. To rectify this omission, we have incorporated the necessary information in the revised manuscript.

Initially, the principle for determining the number of perforations was elucidated. The quantity of BMS perforations is not constant but is dynamically adjusted based on the area of the exposed footprint during surgery. Specifically, larger rotator cuff tears correspond to a greater exposed footprint area, necessitating an increased number of perforations. All perforations are evenly distributed within the footprint area at 5 mm intervals, extending from the edge of the articular cartilage to the lateral crest of the greater tuberosity, with drilling continuing until bone marrow fluid is exuded.

Second, we reported the range of perforation numbers within our patient cohort. Among the 44 patients included in this study, the number of BMS perforations varied from 6 to 12, with the exact number tailored to the size of each patient’s tear and footprint area.

Third, we emphasized critical operational aspects to ensure reproducibility. These include ensuring that perforations encompass the entire footprint area, positioning the lateral row anchors at the distal end of the greater tuberosity to fully cover the BMS holes, using the exudation of bone marrow fluid as the biological endpoint, and avoiding excessive drilling to prevent bone damage.

The aforementioned content has been incorporated into Section 2.2 (Surgical Procedure) of the Methods in the revised manuscript and has been appropriately highlighted. We consider that these additions have enhanced the description of the BMS procedure, rendering it more detailed, quantitative, and reproducible.

5. Statistical analysis
Line206-210
The statistical methods are insufficiently described. Please specify the tests used and whether adjustments for multiple comparisons were performed.

Response:

We sincerely appreciate your identification of the deficiencies in our description of the statistical methods. We concur that comprehensive explanations of statistical procedures are essential to ensure the reliability and reproducibility of research findings. In the original manuscript, the description of the statistical analysis was insufficiently detailed, lacking specification of the exact tests employed and whether adjustments for multiple comparisons were implemented. We have addressed each of your concerns individually and have made the corresponding revisions to the manuscript.

1. Response Concerning the "Tests Used"

The primary analysis of this study entailed comparing clinical indicators obtained before surgery with those collected at a 24-month postoperative follow-up. Given that all measurement indicators constituted paired data from the same cohort of patients at two distinct time points, paired-sample t-tests were employed for statistical evaluation. Specifically, paired comparisons were conducted for the following variables: clinical scores (VAS, SANE and ASES), joint range of motion (FF, ER and IR), and JOA scores, which were measured at three time points preoperatively, 6 months postoperatively, and 24 months postoperatively. For the analysis of JOA scores across these three time points, repeated-measures ANOVA was utilized for overall comparison, followed by pairwise post hoc tests.

2. Response concerning whether adjustments for multiple comparisons were implemented.

Concerning the issue of multiple comparison corrections, we addressed two distinct scenarios separately. For the primary clinical indicators (VAS, SANE, ASES, FF, ER and IR), paired comparisons were conducted solely at a single time point (preoperative versus 24 months postoperative). Consequently, each indicator was tested only once, rendering multiple comparison correction unnecessary 4. In contrast, for the JOA score, which was measured repeatedly at three time points, the Bonferroni correction was applied during post-hoc pairwise comparisons to adjust the significance level and control the cumulative risk of Type I error.

We have incorporated all the aforementioned details into the revised statistical methods section and have accordingly updated the figure legends related to multiple comparisons of JOA. All modifications have been clearly highlighted. We believe that these revisions comprehensively address your concerns and enhance the description of the statistical methods to satisfy reproducibility standards.

6. Critical error in Table 2
Table 2 appears to contain incorrect data (duplicating clinical scores instead of range-of-motion values). This is a major issue that must be corrected.

Response:

We sincerely appreciate the reviewers' thorough and meticulous evaluation. Following a comprehensive, item-by-item verification of the original data records and statistical output files, we wish to provide the following clarifications.

Firstly, the data presented are accurate. All values in Table 2 were obtained from the original medical records and joint range of motion measurements recorded in the outpatient follow-up database, and have been thoroughly re-verified for accuracy. Secondly, the error pertains to a labeling issue. During the preparation of the table, the column headers from Table 1 were inadvertently copied into Table 2, resulting in the display of scoring scale names (ASES/SANE/VAS) instead of the appropriate joint range of motion indicators (FF/ER/IR). This mislabeling led to confusion among the reviewers and constitutes a significant and unacceptable formatting error, for which we offer our sincere apologies.

In this revision, the column headers in Table 2 have been corrected to accurately reflect the joint range of motion indicators (FF, ER, IR) along with their respective units. Additionally, Table 1 has been thoroughly reviewed, confirming that its title, column headers, and data are accurate and require no modifications.

We sincerely apologize once again for the inconvenience caused to the reviewers and editors by this formatting error. We assure you that all tables in this revision have been meticulously reviewed item by item to confirm that the titles accurately correspond to the content and that the data are precise.
________________________________________
Minor comments
• Line193-194 Please provide a reference for the JOA score for international readers.

Response:

We sincerely appreciate the reviewers' valuable suggestions. We acknowledge that the JOA score, developed by the Japanese Orthopaedic Association as a system for evaluating shoulder function, is not as widely recognized internationally as scoring systems such as the ASES and Constant scores. Consequently, it is essential to provide clear scoring references to facilitate understanding among international readers.

The original manuscript referenced the JOA score and enumerated its five components; however, it did not include specific evaluation criteria for each sub-item nor clinical interpretation references. To rectify this, we have incorporated detailed supplementary information regarding the JOA score in Section 2.3 of the Methods section in the revised manuscript, with the additions clearly highlighted. We believe these enhancements will facilitate a clearer understanding of the composition and clinical relevance of the JOA score. We appreciate your valuable suggestions.
• Line101-111 Clarify inclusion criteria (partial vs full-thickness tears).

Response:

We sincerely appreciate the reviewers' thorough and insightful comments. We concur that precise reporting of tear type and size is essential for readers to comprehend the characteristics of the study population. In the original manuscript, patients were described only generally as "preoperatively diagnosed with RCT," without explicit definition of tear type and size, which represents a notable limitation.

In this revision, Section 2.1 of the Methods has been thoroughly refined and enhanced. Firstly, it was clarified that all 44 patients included in the study presented with full-thickness rotator cuff tears, as confirmed by both preoperative magnetic resonance imaging (MRI) and intraoperative arthroscopic examination. Secondly, the classification of tear size was standardized according to the Cofield system, categorizing tears as small (≤1 cm), medium (1–3 cm), large (3–5 cm), or massive (>5 cm). Tear size was determined by measuring the maximum anteroposterior diameter of the tendon on preoperative MRI oblique coronal images. Thirdly, the specific distribution of tear sizes within the cohort was accurately reported: 33 cases (75.0%) were medium tears (including 27 cases measuring 2.0 cm and 6 cases measuring 2.5 cm); 7 cases (15.9%) were large tears (5 cases measuring 3.0 cm and 2 cases measuring 3.5 cm); and 4 cases (9.1%) were massive tears, each measuring 5.0 cm. No small tears (≤1 cm) were included in the study. Finally, the baseline description of tear sizes was supplemented in the Results section to provide a comprehensive overview of the study population’s characteristics.

We contend that the aforementioned additions have rendered the inclusion criteria clear, comprehensive, and reproducible. All modifications have been duly highlighted, and diagnostic information for all patients is provided in the supplementary data. We sincerely appreciate your thorough review and commitment to enhancing the rigor of our manuscript.
• Line 219-220 Define MRI-based healing criteria more clearly.

Response:

We sincerely thank the reviewers for their rigorous and precise comments. We fully agree that the original manuscript's assessment of tendon healing status lacked an objective and standardized classification system, and that using only a general description of "complete or partial healing" is insufficiently standardized in the field of rotator cuff repair research.

In response to this issue, we have re-evaluated and reported all postoperative 24-month MRI images using the Sugaya classification system in the revised manuscript. The Sugaya classification is currently the most widely adopted MRI evaluation system internationally for assessing postoperative rotator cuff repair, categorizing repair status into five types. Type I indicates sufficient rotator cuff thickness with a uniform signal; Type II denotes sufficient thickness with a partial high signal; Type III reflects thinning of the rotator cuff without discontinuity; Type IV corresponds to a small tear in the rotator cuff; and Type V signifies a large tear in the rotator cuff. Types I through III are considered indicative of successful healing, whereas Types IV and V are classified as re-tears.

Two independent radiologists were invited to re-evaluate the postoperative MRIs of all 44 patients using a double-blind methodology. Among these cases, 18 (40.9%) were classified as Type I, 19 (43.2%) as Type II, and 7 (15.9%) as Type III; no cases were identified as Type IV or Type V. Consequently, all 44 patients demonstrated Sugaya Type I–III healing, with no imaging evidence of re-tear. These findings are consistent with the original manuscript’s assertion that "all patients healed." Notably, the current reporting approach employs standardized criteria that are quantifiable, comparable, and reproducible.

We have comprehensively expanded the criteria and evaluation procedures of the Sugaya classification in Section 2.3 of the Methods in the revised manuscript. Additionally, in the Results section, we substituted the prior general descriptions with detailed classification data and made corresponding modifications in the Abstract. All changes have been clearly highlighted. We are confident that these revisions adequately address your concerns and align the assessment of tendon healing with the standardized requirements of the field.

Reviewer #3: This article retrospectively analyzed the postoperative functional and imaging results of 44 patients with rotator cuff tears who underwent the modified Mason-Allen suture bridge (the "parachute") combined with bone marrow stimulation (BMS) technique. The article has a complete structure, detailed description of the surgical technique, and high clinical relevance. However, there are some issues that need to be improved before publication.
1. This study is a single-arm retrospective analysis without a control group, which limits the judgment of the superiority of the "parachute" technique. The study may be affected by selection bias and confounding factors (such as tear size, tendon quality, and patient comorbidities). It is recommended that the authors clearly state the limitations of the conclusion in the methods and discussion sections.

Response:

We sincerely appreciate the reviewers for identifying this fundamental methodological issue. We fully concur with their perspectives and have incorporated comprehensive responses and corresponding revisions in the updated manuscript.

First, concerning the absence of a control group, we have revised the overall tone of the conclusions. Statements in the original manuscript that suggested superiority, such as "this technique provides a safe and effective treatment for rotator cuff repair," have been modified to more objective expressions, for example, "this technique is a safe and feasible treatment option." Furthermore, we have explicitly indicated multiple times throughout the abstract, introduction, discussion, and conclusion that this study is an exploratory case series and that its findings do not constitute confirmatory evidence of efficacy.

Second, regarding selection bias and confounding factors, we have supplemented the revised manuscript with comprehensive baseline characteristics to enable readers to clearly understand the composition of the study population. In the limitations section of the discussion, we explicitly enumerate potential confounding factors that may influence the results, including tear size, tendon quality, patient age, comorbidities, and rehabilitation compliance. We also candidly acknowledge that this study is unable to adjust for these confounders through multivariate regression or propensity score matching.

Third, concerning the study's positioning, we have explicitly characterized this research in the revised manuscript as a "hypothesis-generating exploratory study" rather than a confirmatory investigation. All discussions pertaining to the underlying mechanisms of the technique are identified as theoretical inferences that necessitate validation through future research.

Fourth, concerning future directions, the conclusion explicitly calls for prospective randomized controlled trials to directly compare the efficacy of the "parachute" technique with traditional procedures and to delineate the independent effects of each intervention component.

We believe that the aforementioned revisions have comprehensively addressed your concerns, thereby enhancing the accuracy of the study’s positioning and the rigor of its conclusions. All modifications have been clearly highlighted. We sincerely appreciate your thorough evaluation of the manuscript’s methodological quality.

2. The post-MRI assessment is limited, and there are no criteria for "complete healing" and "partial healing". It is recommended to clarify the MRI criteria and explain the basis for the determination of partial healing and complete healing.

Response:

We sincerely thank the reviewers for their rigorous and precise comments. We fully agree that the original manuscript's assessment of tendon healing status lacked an objective and standardized classification system, and that using only a general description of "complete or partial healing" is insufficiently standardized in the field of rotator cuff repair research.

In response to this issue, we have re-evaluated and reported all postoperative 24-month MRI images using the Sugaya classification system in the revised manuscript. The Sugaya classification is currently the most widely adopted MRI evaluation system internationally for assessing postoperative rotator cuff repair, categorizing repair status into five types. Type I indicates sufficient rotator cuff thickness with a uniform signal; Type II denotes sufficient thickness with a partial high signal; Type III reflects thinning of the rotator cuff without discontinuity; Type IV corresponds to a small tear in the rotator cuff; and Type V signifies a large tear in the rotator cuff. Types I through III are considered indicative of successful healing, whereas Types IV and V are classified as re-tears.

Two independent radiologists were invited to re-evaluate the postoperative MRIs of all 44 patients using a double-blind methodology. Among these cases, 18 (40.9%) were classified as Type I, 19 (43.2%) as Type II, and 7 (15.9%) as Type III; no cases were identified as Type IV or Type V. Consequently, all 44 patients demonstrated Sugaya Type I–III healing, with no imaging evidence of re-tear. These findings are consistent with the original manuscript’s assertion that "all patients healed." Notably, the current reporting approach employs standardized criteria that are quantifiable, comparable, and reproducible.

We have comprehensively expanded the criteria and evaluation procedures of the Sugaya classification in Section 2.3 of the Methods in the revised manuscript. Additionally, in the Results section, we substituted the prior general descriptions with detailed classification data and made corresponding modifications in the Abstract. All changes have been clearly highlighted. We are confident that these revisions adequately address your concerns and align the assessment of tendon healing with the standardized requirements of the field.

3. No subgroup analysis was conducted based on tear size, age, or tendon quality, making it difficult to determine which patients are most suitable for this technique. It is recommended that the authors conduct subgroup analysis in this section and discuss the results.

Response:

We sincerely appreciate your constructive comments. We concur that subgroup analysis is crucial for identifying the optimal patient population for this technique. However, given the specific circumstances of this study, we are unable to perform a reliable subgroup analysis for the following reasons.

First, the sample size in this study is limited, leading to substantially insufficient statistical power. A total of 44 patients were included. According to the Cofield classification, 33 cases (75.0%) involved medium-sized tears, 7 cases (15.9%) involved large tears, and 4 cases (9.1%) involved massive tears. The small sample sizes within these subgroups preclude meaningful statistical comparisons.

Second, the divisions of age subgroups lack a standardized criterion. At present, no universally accepted cutoff values for age stratification following rotator cuff repair exist in the literature. The arbitrary categorization of age subgroups in this study is not supported by evidence-based rationale and may result in unreliable conclusions.

Third, the distribution of Goutallier grades was limited. Because this study excluded patients with grades of 3 or higher, all participants exhibited fatty infiltration levels ranging only from 0 to 2. Consequently, the study population was relatively homogeneous, precluding subgroup analyses based on tendon quality. Although this design effectively controlled for tendon quality as a confounding variable, it also precluded examination of the potential moderating effect of tendon quality on technical outcomes.

Fourth, the original design of this study did not aim to detect differences among subgroups. Subgroup analyses should be grounded in predefined research hypotheses and supported by adequate sample sizes. Given its exploratory nature, this study is not appropriate for post hoc subgroup analyses.

Nevertheless, these limitations have been explicitly addressed in the discussion section of the revised manuscript, where we also underscore the necessity for future prospective studies with larger sample sizes to establish the optimal indications for this technique. We contend that this transparent acknowledgment of limitations will facilitate a more accurate interpretation of the study findings by readers.

4. Figures 6 (MRI) and Figures 2–4 (surgical steps) are helpful for understanding, but some images are not clear enough regarding the healing status or node position.

Response:

We sincerely appreciate your recognition of Figures 2-4 and 6, as well as your constructive feedback regarding the image clarity. We fully concur with your observations and have taken careful measures to address these issues.

In this revision, all images have been replaced with high-resolution versions. Additionally, the figure captions have been meticulously reviewed to ensure that the labeling information precisely corresponds with the descriptions provided in the text, thereby enhancing readers' comprehension.

We believe that the substituted high-definition images more effectively illustrate the critical surgical techniques and the postoperative healing process. Should you have any additional requirements concerning image format or resolution, we remain prepared to make adjustments as needed.

5. The terms "continuous RCT" in the abstract are not clear. It is recommended to correct them, and similar issues exist throughout the article. It is recommended to uniformly correct them.

Response:

We sincerely appreciate your attention to the issue of inaccurate terminology usage. We concur that the term "continuous RCT" is ambiguous and susceptible to misinterpretation. Our initial intention was to refer to patients with rotator cuff tears who remained continuously in the study group for 24 months; however, this phrasing was potentially confusing or ambiguous. Consequently, based on your recommendation, we have removed this term from the abstract. Additionally, we have reviewed the entire manuscript to identify and correct any similar issues. These revisions are highlighted in yellow in the updated manuscript. We appreciate your careful guidance.

6. The sample size of this article is only 44 cases, and it is a single-center study. It is recommended to explain whether the results are generalizable.

Response:

We sincerely appreciate your attention to this significant issue. We concur that the limited sample size and single-center design present constraints on the generalizability of the study findings. In response, we have provided a detailed explanation of this matter and have incorporated the corresponding revisions into the manuscript.

Firstly, an objective explanation concerning the sample size is warranted. This study included patients who underwent the specified treatment technique and met stringent inclusion criteria between January 2021 and January 2023. As a single-center report conducted during the initial implementation phase of a novel technology, 44 cases completed full follow-up within a two-year period. This sample size is considered appropriate for exploratory research at this stage. Concurrently, the application of rigorous exclusion criteria (e.g., Goutallier grade ≥3, prior surgical history, arthritis) ensured population homogeneity but consequently limited the potential for further sample size expansion.

Second, concerning the rationale for employing a single-center design, a primary objective of this study was to ensure standardization and consistency in the surgical technique during the initial implementation of the new technology. This approach aimed to minimize confounding bias arising from inter-operator variability. Consequently, the use of a single-center design is justified at this stage; however, it necessitates cautious interpretation regarding the external generalizability of the findings.

Third, concerning the generalizability of the findings, we posit that the results of this study are directly applicable to a patient population aged 35 to 80 years presenting with small to large full-thickness rotator cuff tears (2–5 cm), classified as Goutallier grade 0 to 2, without prior surgical history or severe shoulder arthritis, and who undergo surgery performed by experienced shoulder arthroscopy surgeons at specialized centers. However, for patients with smaller or larger tears, Goutallier grade ≥3, complex comorbidities, or those treated in clinical settings with less surgical expertise, the applicability of the study’s conclusions may be limited.

Fourth, concerning future directions, we have explicitly included a discussion on the issue of generalizability in the limitations section of the revised manuscript. We also emphasized that future validation of the applicability of this technique to a broader population necessitates multicenter studies with large sample sizes.

Although the sample size in this study is relatively small, the comprehensive baseline data, including tear type, size, location, and Goutallier grading, offer readers adequate information to assess the external validity of the findings.

7. It is recommended that the authors carefully check the citations of references to avoid duplicate citations.

We sincerely appreciate your thorough and meticulous evaluation. In response to your suggestions, we have carefully examined all references cited in the manuscript. During this verification process, we identified two instances of duplicate citations, which have been removed. Subsequently, all citations have been renumbered to ensure a one-to-one correspondence between in-text citations and the reference list. No additional duplicate citations were detected upon further review. We greatly appreciate your rigorous attention to detail regarding the references.
