# Supplementary material for: Retrospective analysis of arthroscopic repair outcomes using a modified Mason-Allen suture bridge “parachute” technique with bone marrow stimulation for rotator cuff tears
Source: PLoS One. 2026 Aug 20;21(8):e0356245. doi: 10.1371/journal.pone.0356245 (PMC13492772; doi:10.1371/journal.pone.0356245)
Supplement: Peer review history

# Peer review: Decision Letter 0

**DOI:** 10.1371/journal.pone.0356245.r001

**Authors:** Emil George Haritinian

17 Jun 2026

Dear Dr. Dou,

Thank you for submitting your manuscript to PLOS ONE. After careful consideration, we feel that it has merit but does not fully meet PLOS ONE’s publication criteria as it currently stands. Therefore, we invite you to submit a revised version of the manuscript that addresses the points raised during the review process.

==============================

The topic is clinically relevant, and the technique you describe is of interest to shoulder surgeons. However, after careful review, the manuscript is not suitable for publication in its current form.

Several major issues that must be addressed before the paper can be reconsidered. The most important concerns relate to study design, missing methodological details, and unclear reporting. Key elements of patient selection, tear characteristics, tendon quality, and rehabilitation are not described. The definitions of healing and retear are unclear, and the MRI criteria are not standardized. The description of the bone marrow stimulation procedure lacks essential quantitative details. Statistical methods are not reported, and Table 2 contains clear errors that must be corrected.

A major limitation of the study is the absence of a control group, which makes it difficult to interpret the effectiveness of the combined technique. The discussion also overstates the conclusions and makes claims that are not supported by the data. Reviewers also noted issues with English language clarity, duplicated references, and inconsistent terminology.

Given these substantial concerns, the manuscript requires major revision. If you choose to revise the paper, please address all reviewer comments thoroughly and provide a clearer, more transparent presentation of your methods, results, and limitations.

==============================

Please submit your revised manuscript by Aug 01 2026 11:59PM. If you will need more time than this to complete your revisions, please reply to this message or contact the journal office at plosone@plos.org. When you're ready to submit your revision, log on to https://www.editorialmanager.com/pone/ and select the 'Submissions Needing Revision' folder to locate your manuscript file.

- A letter that responds to each point raised by the academic editor and reviewer(s). You should upload this letter as a separate file labeled 'Response to Reviewers'.
- A marked-up copy of your manuscript that highlights changes made to the original version. You should upload this as a separate file labeled 'Revised Manuscript with Track Changes'.
- An unmarked version of your revised paper without tracked changes. You should upload this as a separate file labeled 'Manuscript'.

If you would like to make changes to your financial disclosure, please include your updated statement in your cover letter. Guidelines for resubmitting your figure files are available below the reviewer comments at the end of this letter.

If applicable, we recommend that you deposit your laboratory protocols in protocols.io to enhance the reproducibility of your results. Protocols.io assigns your protocol its own identifier (DOI) so that it can be cited independently in the future. For instructions see: https://journals.plos.org/plosone/s/submission-guidelines#loc-laboratory-protocols. Additionally, PLOS ONE offers an option for publishing peer-reviewed Lab Protocol articles, which describe protocols hosted on protocols.io. Read more information on sharing protocols at https://plos.org/protocols?utm_medium=editorial-email&utm_source=authorletters&utm_campaign=protocols.

As the corresponding author, your ORCID iD is verified in the submission system and will appear in the published article. PLOS supports the use of ORCID, and we encourage all coauthors to register for an ORCID iD and use it as well. Please encourage your coauthors to verify their ORCID iD within the submission system before final acceptance, as unverified ORCID iDs will not appear in the published article. *Only*  the individual author can complete the verification step; PLOS staff *cannot*  verify ORCID iDs on behalf of authors.

We look forward to receiving your revised manuscript.

Kind regards,

Emil George Haritinian, M.D, Ph.D.

Academic Editor

PLOS One

Journal Requirements:

When submitting your revision, we need you to address these additional requirements.

1. Please ensure that your manuscript meets PLOS ONE's style requirements, including those for file naming. The PLOS ONE style templates can be found at https://journals.plos.org/plosone/s/file?id=wjVg/PLOSOne_formatting_sample_main_body.pdf and https://journals.plos.org/plosone/s/file?id=ba62/PLOSOne_formatting_sample_title_authors_affiliations.pdf

2. Thank you for stating in your Funding Statement:

[This research Supported by Medical and Health Scientific Research Project of Qingdao (2024-WJKY060).].

Please provide an amended statement that declares *all* the funding or sources of support (whether external or internal to your organization) received during this study, as detailed online in our guide for authors at http://journals.plos.org/plosone/s/submit-now.  Please also include the statement “There was no additional external funding received for this study.” in your updated Funding Statement.

Please include your amended Funding Statement within your cover letter. We will change the online submission form on your behalf.

3. Please remove any funding-related text from the manuscript. Please note that funding information should not appear in any section or other areas of your manuscript. We will publish the funding statement that is present in the Financial Disclosure section of the online submission form.

4. In the online submission form, you indicated that [The data of this study are available from the corresponding author upon reasonable request.].

All PLOS journals now require all data underlying the findings described in their manuscript to be freely available to other researchers, either 1. In a public repository, 2. Within the manuscript itself, or 3. Uploaded as supplementary information.

This policy applies to all data except where public deposition would breach compliance with the protocol approved by your research ethics board. If your data cannot be made publicly available for ethical or legal reasons (e.g., public availability would compromise patient privacy), please explain your reasons on resubmission and your exemption request will be escalated for approval.

5. PLOS requires an ORCID iD for the corresponding author in Editorial Manager on papers submitted after December 6th, 2016. Please ensure that you have an ORCID iD and that it is validated in Editorial Manager. To do this, go to ‘Update my Information’ (in the upper left-hand corner of the main menu), and click on the Fetch/Validate link next to the ORCID field. This will take you to the ORCID site and allow you to create a new iD or authenticate a pre-existing iD in Editorial Manager.

6. We note that Figure 1 in your submission contains images which may be copyrighted. All PLOS content is published under the Creative Commons Attribution License (CC BY 4.0), which means that the manuscript, images, and Supporting Information files will be freely available online, and any third party is permitted to access, download, copy, distribute, and use these materials in any way, even commercially, with proper attribution. For more information, see our copyright guidelines: http://journals.plos.org/plosone/s/licenses-and-copyright.

We require you to either (1) present written permission from the copyright holder to publish these figures specifically under the CC BY 4.0 license, or (2) remove the figures from your submission:

1. You may seek permission from the original copyright holder of Figure 1 to publish the content specifically under the CC BY 4.0 license.

We recommend that you contact the original copyright holder with the Content Permission Form (http://journals.plos.org/plosone/s/file?id=7c09/content-permission-form.pdf) and the following text:

“I request permission for the open-access journal PLOS ONE to publish XXX under the Creative Commons Attribution License (CCAL) CC BY 4.0 (http://creativecommons.org/licenses/by/4.0/). Please be aware that this license allows unrestricted use and distribution, even commercially, by third parties. Please reply and provide explicit written permission to publish XXX under a CC BY license and complete the attached form.”

Please upload the completed Content Permission Form or other proof of granted permissions as an "Other" file with your submission.

In the figure caption of the copyrighted figure, please include the following text: “Reprinted from [ref] under a CC BY license, with permission from [name of publisher], original copyright [original copyright year].”

2. If you are unable to obtain permission from the original copyright holder to publish these figures under the CC BY 4.0 license or if the copyright holder’s requirements are incompatible with the CC BY 4.0 license, please either i) remove the figure or ii) supply a replacement figure that complies with the CC BY 4.0 license. Please check copyright information on all replacement figures and update the figure caption with source information. If applicable, please specify in the figure caption text when a figure is similar but not identical to the original image and is therefore for illustrative purposes only.

If the reviewer comments include a recommendation to cite specific previously published works, please review and evaluate these publications to determine whether they are relevant and should be cited. There is no requirement to cite these works unless the editor has indicated otherwise.

[Note: HTML markup is below. Please do not edit.]

Reviewers' comments:

Reviewer's Responses to Questions

**Comments to the Author**

1. Is the manuscript technically sound, and do the data support the conclusions?

Reviewer #1: Partly

Reviewer #2: No

Reviewer #3: Yes

**********

2. Has the statistical analysis been performed appropriately and rigorously?

Reviewer #1: No

Reviewer #2: No

Reviewer #3: Yes

**********

3. Have the authors made all data underlying the findings in their manuscript fully available?

The PLOS Data policy

Reviewer #1: Yes

Reviewer #2: Yes

Reviewer #3: Yes

**********

4. Is the manuscript presented in an intelligible fashion and written in standard English?

Reviewer #1: Yes

Reviewer #2: Yes

Reviewer #3: Yes

**********

Reviewer #1: This paper discusses the management of rotator cuff tears with a unique surgical technique. This surgical technique combines a modified Mason Allen suture bridge with bone marrow stimulation. The topic is clinically relevant, and the authors report good clinical outcomes and structural healing at an extended follow up period of 24 months. However, in its current form the manuscript has major methodological and reporting limitations that need to be addressed.

It is unclear what patient population this study has been conducted in as the specific tear (i.e. supraspinatus), tear size or tear type (partial or full thickness) has not been reported. Furthermore, the paper reports that the presence of rotator cuff lesions was an exclusion criteria. It is unclear what these lesions refer to. Furthermore, the authors should clearly report on tendon quality given Goutallier grade 3 or higher fatty infiltration was excluded. Concomitant procedures should also be reported alongside number of anchors used. Rehabilitation protocols should also be reported.

Minor comments:

The manuscript would benefit from substantial English language editing. There are frequent grammatical issues, awkward phrasing, and terminology inconsistencies that reduce readability.

The reference list contains duplicated references including for example 4 and 9. 10 and 11.

Introduction:

The introduction is straightforward. The manuscript would benefit from discussing retear rates for bone marrow stimulation in cuff repairs. Second, the introduction blends technical description with anticipated benefits in a way that appears promotional rather than scientific. Given this is a retrospective case serious, the introduction should frame this as an evaluation of the technique in practice.

Methods:

The methods section requires major revision. Inclusion and exclusion criteria require clarification. Specifically, tear characteristics need to be clearly recorded. This includes full thickness vs partial thickness tears, tear size, tear location (in which rotator cuff tendon). Furthermore, the methods describes exclusion of patients with rotator cuff lesions which is inconsistent given this is a population with rotator cuff tears.

It is unclear how it was determined that repairs were intact or ‘healing’. Please report using a standardised classification such as Sugaya.

Statistical analysis methods were not reported on. It is unclear what statistical tests were used to determine significant results. This must be reported.

Results:

The results section does not include key patient characteristics as outlined in comments above. Furthermore Table 2 is incorrectly labelled. The values suggest it is range of motion though it is labelled ASES, SANE and VAS.

Discussion:

The discussion currently significantly overstates the implications of the data and makes mechanistic claims. Whilst it is biologically plausible that the parachute structure can enhance sealing and bone marrow leakage, it is unclear whether this is true as it is not within the scope of the study and not mechanistically proven. Furthermore, reported mechanical benefits of the parachute technique can be suggested but they remain unproven and not within the scope of the results of this paper. The manuscript should suggest that this repair method supports feasibility rather than superiority compared to other techniques.

Reviewer #2: Reviewer Comments

I would like to thank the Editor for the opportunity to review this manuscript. The topic is clinically relevant, and the authors present an interesting surgical approach. However, I have several major concerns regarding the study design and reporting.

The most important limitation is the absence of a control group. Because the intervention combines a modified Mason-Allen “Parachute” technique and bone marrow stimulation (BMS), it is not possible to determine which component contributes to the reported outcomes. In addition, key information regarding patient characteristics and tear morphology is insufficient, and there are concerns about data presentation (e.g., Table 2).

Overall, the current manuscript does not provide sufficient evidence to support the authors’ conclusions.

Major comments

1. Lack of a control group

The most critical limitation is the absence of a control or comparison group. Because the intervention combines a modified Mason-Allen “Parachute” technique and BMS, it is unclear whether the favorable outcomes are attributable to the repair configuration, BMS, or patient selection. Therefore, the main conclusion is not sufficiently supported.

2. Insufficient patient and tear characterization

Line101-111

Key baseline information is missing, including:

• partial vs full-thickness tears,

• involved tendons (e.g., supraspinatus/infraspinatus/subscapularis),

• tear size and classification,

• Detailed Goutallier grade distribution.

Without these details, the reported healing rate cannot be appropriately interpreted.

3. Unclear definitions (healing and retear)

Line34

The definitions of “complete healing,” “partial healing,” and “retear” are not provided. Clear, reproducible criteria are required.

4. Inadequate description of BMS procedure

Line144-147

The number of perforations for BMS is not reported. More detailed and quantitative description is necessary for reproducibility.

5. Statistical analysis

Line206-210

The statistical methods are insufficiently described. Please specify the tests used and whether adjustments for multiple comparisons were performed.

6. Critical error in Table 2

Table 2 appears to contain incorrect data (duplicating clinical scores instead of range-of-motion values). This is a major issue that must be corrected.

________________________________________

Minor comments

• Line193-194 Please provide a reference for the JOA score for international readers.

• Line101-111 Clarify inclusion criteria (partial vs full-thickness tears).

• Line 219-220 Define MRI-based healing criteria more clearly.

Reviewer #3: This article retrospectively analyzed the postoperative functional and imaging results of 44 patients with rotator cuff tears who underwent the modified Mason-Allen suture bridge (the "parachute") combined with bone marrow stimulation (BMS) technique. The article has a complete structure, detailed description of the surgical technique, and high clinical relevance. However, there are some issues that need to be improved before publication.

1. This study is a single-arm retrospective analysis without a control group, which limits the judgment of the superiority of the "parachute" technique. The study may be affected by selection bias and confounding factors (such as tear size, tendon quality, and patient comorbidities). It is recommended that the authors clearly state the limitations of the conclusion in the methods and discussion sections.

2. The post-MRI assessment is limited, and there are no criteria for "complete healing" and "partial healing". It is recommended to clarify the MRI criteria and explain the basis for the determination of partial healing and complete healing.

3. No subgroup analysis was conducted based on tear size, age, or tendon quality, making it difficult to determine which patients are most suitable for this technique. It is recommended that the authors conduct subgroup analysis in this section and discuss the results.

4. Figures 6 (MRI) and Figures 2–4 (surgical steps) are helpful for understanding, but some images are not clear enough regarding the healing status or node position.

5. The terms "continuous RCT" in the abstract are not clear. It is recommended to correct them, and similar issues exist throughout the article. It is recommended to uniformly correct them.

6. The sample size of this article is only 44 cases, and it is a single-center study. It is recommended to explain whether the results are generalizable.

7. It is recommended that the authors carefully check the citations of references to avoid duplicate citations.

**********

what does this mean?). If published, this will include your full peer review and any attached files.

If you choose “no”, your identity will remain anonymous but your review may still be made public.

**Do you want your identity to be public for this peer review?**  For information about this choice, including consent withdrawal, please see our Privacy Policy

Reviewer #1: No

Reviewer #2: **Yes:** Terufumi Shibata

Reviewer #3: No

**********

[NOTE: If reviewer comments were submitted as an attachment file, they will be attached to this email and accessible via the submission site. Please log into your account, locate the manuscript record, and check for the action link "View Attachments". If this link does not appear, there are no attachment files.]

To ensure your figures meet our technical requirements, please review our figure guidelines: https://journals.plos.org/plosone/s/figures

You may also use PLOS’s free figure tool, NAAS, to help you prepare publication quality figures: https://journals.plos.org/plosone/s/figures#loc-tools-for-figure-preparation.

NAAS will assess whether your figures meet our technical requirements by comparing each figure against our figure specifications.

# Author comment: Author response to Decision Letter 1

**DOI:** 10.1371/journal.pone.0356245.r002

13 Jul 2026

Reviewer #1: This paper discusses the management of rotator cuff tears with a unique surgical technique. This surgical technique combines a modified Mason Allen suture bridge with bone marrow stimulation. The topic is clinically relevant, and the authors report good clinical outcomes and structural healing at an extended follow up period of 24 months. However, in its current form the manuscript has major methodological and reporting limitations that need to be addressed.

It is unclear what patient population this study has been conducted in as the specific tear (i.e. supraspinatus), tear size or tear type (partial or full thickness) has not been reported. Furthermore, the paper reports that the presence of rotator cuff lesions was an exclusion criteria. It is unclear what these lesions refer to. Furthermore, the authors should clearly report on tendon quality given Goutallier grade 3 or higher fatty infiltration was excluded. Concomitant procedures should also be reported alongside number of anchors used. Rehabilitation protocols should also be reported.

Response:

We sincerely appreciate your thorough and detailed comments, which have been instrumental in enhancing the manuscript's comprehensiveness and reproducibility. Each point has been addressed meticulously.

First, concerning the tear characteristics of the patient cohort, the revised manuscript has been supplemented with a comprehensive description of the tear features. All 44 patients in this group presented with full-thickness rotator cuff tears, classified according to the Cofield system as medium-sized in 33 cases (75.0%), large in 7 cases (15.9%), and massive in 4 cases (9.1%). In all patients, the supraspinatus tendon was involved, with some tears extending to the infraspinatus tendon.

Second, regarding the clarification of the exclusion criteria for "rotator cuff lesions," the original statement "excluding rotator cuff lesions" was inaccurate and logically inconsistent with the study population, which comprised patients with rotator cuff tears. The intended exclusion criteria were other lesions that could confound shoulder joint function, such as shoulder instability, frozen shoulder, or sequelae of fractures. This has been rectified in the revised manuscript, where these specific conditions are explicitly enumerated.

Third, concerning the reporting of tendon quality, since patients with a Goutallier grade of 3 or higher were excluded, it is essential to provide the distribution of Goutallier grades among the enrolled patients. This information has been incorporated into the baseline characteristics table in the revised manuscript.

Fourth, concerning the number of anchors utilized, the revised manuscript has been supplemented with detailed information regarding the anchor configuration.

Fifth, regarding the rehabilitation protocol, we have elaborated the description in the Methods section by transforming the initial brief overview into a detailed, staged standardized rehabilitation protocol. This includes specifications of activity restrictions, allowed movements, and progression schedules at each stage, thereby enhancing its utility as a reference for other clinical centers.

All the aforementioned modifications have been highlighted in yellow in the revised manuscript. We believe that these additions have enhanced the completeness, clarity, and reproducibility of the reporting of the study population characteristics in accordance with academic standards.

Minor comments:

The manuscript would benefit from substantial English language editing. There are frequent grammatical issues, awkward phrasing, and terminology inconsistencies that reduce readability.

The reference list contains duplicated references including for example 4 and 9. 10 and 11.

Response:

We sincerely appreciate your valuable suggestions for improvement and fully acknowledge your criticisms. The original manuscript contained issues related to grammar, awkward phrasing, and inconsistent terminology, which adversely affected its overall readability. Furthermore, the references necessitated additional verification and refinement. We have systematically addressed both of these concerns.

Following this revision, we engaged a professional peer with expertise in editing English for medical journals to perform a final review of the manuscript, ensuring that the language adheres to publication standards. Additionally, we meticulously examined the reference list, identified and rectified duplicate citations, and reordered all citation numbers to guarantee precise correspondence between in-text citations and the reference list. We sincerely appreciate your careful attention to the details of the references.

Introduction:

The introduction is straightforward. The manuscript would benefit from discussing retear rates for bone marrow stimulation in cuff repairs. Second, the introduction blends technical description with anticipated benefits in a way that appears promotional rather than scientific. Given this is a retrospective case serious, the introduction should frame this as an evaluation of the technique in practice.

Response:

We sincerely appreciate the reviewers’ valuable comments. We concur that a comprehensive discussion regarding the impact of bone marrow stimulation (BMS) techniques on retear rates in the introduction is essential to substantiate the biological rationale underlying the combined technique employed in this study. In the original manuscript, BMS was only briefly mentioned, and quantitative clinical evidence demonstrating its effect on reducing retear rates was not adequately provided, which represents a notable limitation. We also acknowledge and accept the reviewers’ critique concerning the use of promotional language in the introduction, which is inappropriate for a retrospective observational study. The primary objective of this study is not to advocate for the superiority of a novel technique but rather to objectively assess its performance in terms of safety, feasibility, and early functional recovery based on real-world clinical data, as well as to identify its potential advantages and limitations.

To address these concerns, we have considerably expanded and enhanced the introduction by incorporating recent high-quality systematic reviews and meta-analyses, with explicit citations of data from other researchers. This body of evidence robustly supports that the combined application of BMS in rotator cuff repair can significantly reduce postoperative retear rates, thereby establishing a strong evidence-based medical rationale for selecting this technique. Furthermore, in response to your recommendations, we have revised the concluding paragraph of the introduction by eliminating speculative and promotional language and adopting a more neutral and objective scientific tone to accurately convey the original intent of this retrospective study design.

We sincerely thank the reviewers for their thorough evaluation of this manuscript once again. All the aforementioned revisions have been incorporated into the revised version and are clearly highlighted. We believe that these modifications have comprehensively addressed the reviewers' comments and have substantially improved the scientific rigor and accuracy of the paper. Should there be any further suggestions, we remain willing to make additional improvements.

Methods:

The methods section requires major revision. Inclusion and exclusion criteria require clarification. Specifically, tear characteristics need to be clearly recorded. This includes full thickness vs partial thickness tears, tear size, tear location (in which rotator cuff tendon). Furthermore, the methods describes exclusion of patients with rotator cuff lesions which is inconsistent given this is a population with rotator cuff tears.

It is unclear how it was determined that repairs were intact or ‘healing’. Please report using a standardised classification such as Sugaya.

Statistical analysis methods were not reported on. It is unclear what statistical tests were used to determine significant results. This must be reported.

Response:

We sincerely appreciate the reviewer's valuable suggestions. We will address each of your comments systematically and in detail.

1.Inclusion and exclusion criteria

We sincerely appreciate your insightful critique and detailed guidance concerning the methodology section. We fully acknowledge the validity of your comments. The original manuscript’s methodology section exhibited significant deficiencies: firstly, the inclusion criteria did not adequately specify tear characteristics; secondly, there was a logical inconsistency regarding the exclusion of patients with rotator cuff lesions. We will address each point individually and outline the corresponding revisions implemented.

First, the study population consisted of patients with rotator cuff tears; however, the exclusion of "rotator cuff lesions" presents a logical inconsistency. This issue arose from a translation and expression error. The original intent was to exclude other rotator cuff-related pathologies that could confound the outcomes, aside from the rotator cuff tear itself. Nevertheless, the term "rotator cuff lesions" is imprecise and may be easily mistaken for "rotator cuff tears." In the revised version, this contradictory statement has been removed, and the inclusion and exclusion criteria have been reorganized to reflect a clear and logical hierarchy.

Second, concerning the type of tear, a thorough review of the original medical records and intraoperative notes for all 44 patients in our cohort confirmed that each presented with full-thickness rotator cuff tears; no cases of partial tears were included. This criterion was explicitly specified in the inclusion criteria.

Third, with respect to tear size, the measurement methods and classification standards have been clearly documented in the revised manuscript.

Fourth, concerning the location of the tears, we have enhanced the description of their anatomical sites in the revised manuscript. Specifically, 33 cases involved isolated supraspinatus tendon tears, while 11 cases involved combined tears of the supraspinatus and infraspinatus tendons. Corresponding baseline tables have also been incorporated into the Results section.

In the revised manuscript, we have systematically revised the inclusion and exclusion criteria presented in Section 2.1 of the Methods. Concurrently, we have updated the baseline description in the Results section. We believe that these comprehensive revisions adequately address your concerns, resulting in a description within the Methods section that is clear, complete, and logically coherent.

2.Criteria for Healing

We sincerely thank the reviewers for their rigorous and precise comments. We fully agree that the original manuscript's assessment of tendon healing status lacked an objective and standardized classification system, and that using only a general description of "complete or partial healing" is insufficiently standardized in the field of rotator cuff repair research.

In response to this issue, we have re-evaluated and reported all postoperative 24-month MRI images using the Sugaya classification system in the revised manuscript. The Sugaya classification is currently the most widely adopted MRI evaluation system internationally for assessing postoperative rotator cuff repair, categorizing repair status into five types. Type I indicates sufficient rotator cuff thickness with a uniform signal; Type II denotes sufficient thickness with a partial high signal; Type III reflects thinning of the rotator cuff without discontinuity; Type IV corresponds to a small tear in the rotator cuff; and Type V signifies a large tear in the rotator cuff. Types I through III are considered indicative of successful healing, whereas Types IV and V are classified as re-tears.

Two independent radiologists were invited to re-evaluate the postoperative MRIs of all 44 patients using a double-blind methodology. Among these cases, 18 (40.9%) were classified as Type I, 19 (43.2%) as Type II, and 7 (15.9%) as Type III; no cases were identified as Type IV or Type V. Consequently, all 44 patients demonstrated Sugaya Type I–III healing, with no imaging evidence of re-tear. These findings are consistent with the original manuscript’s assertion that "all patients healed." Notably, the current reporting approach employs standardized criteria that are quantifiable, comparable, and reproducible.

We have comprehensively expanded the criteria and evaluation procedures of the Sugaya classification in Section 2.3 of the Methods in the revised manuscript. Additionally, in the Results section, we substituted the prior general descriptions with detailed classification data and made corresponding modifications in the Abstract. All changes have been clearly highlighted. We are confident that these revisions adequately address your concerns and align the assessment of tendon healing with the standardized requirements of the field.

3.For Statistical analysis.

We sincerely appreciate your identification of the deficiencies in our description of the statistical methods. We concur that a detailed and precise explanation of statistical procedures is essential to ensure the reproducibility of research findings. In the original manuscript, the description of the specific statistical analyses was insufficiently detailed and lacked clear specification of the exact tests applied to each variable, as well as the correction methods employed for multiple comparisons. In response to your valuable feedback, we have extensively revised and expanded the statistical methods section in the updated manuscript.

The primary analysis of this study entailed comparing clinical indicators obtained before surgery with those collected at a 24-month postoperative follow-up. Given that all measurement indicators constituted paired data from the same cohort of patients at two distinct time points, paired-sample t-tests were employed for statistical evaluation. Specifically, paired comparisons were conducted for the following variables: clinical scores (VAS, SANE and ASES), joint range of motion (FF, ER and IR), and JOA scores, which were measured at three time points preoperatively, 6 months postoperatively, and 24 months postoperatively. For the analysis of JOA scores across these three time points, repeated-measures ANOVA was utilized for overall comparison, followed by pairwise post hoc tests.

Concerning the issue of multiple comparison corrections, we addressed two distinct scenarios separately. For the primary clinical indicators (VAS, SANE, ASES, FF, ER and IR), paired comparisons were conducted solely at a single time point (preoperative versus 24 months postoperative). Consequently, each indicator was tested only once, rendering multiple comparison correction unnecessary 4. In contrast, for the JOA score, which was measured repeatedly at three time points, the Bonferroni correction was applied during post-hoc pairwise comparisons to adjust the significance level and control the cumulative risk of Type I error.

We have incorporated all the aforementioned details into the revised statistical methods section and have accordingly updated the figure legends related to multiple comparisons of JOA. All modifications have been clearly highlighted. We believe that these revisions comprehensively address your concerns and enhance the description of the statistical methods to satisfy reproducibility standards.

Results:

The results section does not include key patient characteristics as outlined in comments above. Furthermore Table 2 is incorrectly labelled. The values suggest it is range of motion though it is labelled ASES, SANE and VAS.

Response:

We sincerely thank you for valuable feedback. We acknowledge that the original manuscript's results section included only basic demographic information, such as age and gender, and omitted critical baseline lesion characteristics, including tear type, tear size, and tear location. This omission represents a significant gap in the reporting of results. We have thoroughly addressed this issue in the current revision. Initially, we reported the tear characteristics of all 44 patients in the results section, detailing tear typ

Attachment Submitted filename: Response to Reviewers.docx

# Peer review: Decision Letter 1

**DOI:** 10.1371/journal.pone.0356245.r003

**Authors:** Emil George Haritinian

3 Aug 2026

Retrospective Analysis of Arthroscopic Repair Outcomes Using a Modified Mason-Allen Suture Bridge “Parachute” Technique With Bone Marrow Stimulation for Rotator Cuff Tears

PONE-D-26-16987R1

Dear Dr. Dou,

We’re pleased to inform you that your manuscript has been judged scientifically suitable for publication and will be formally accepted for publication once it meets all outstanding technical requirements.

Within one week, you’ll receive an e-mail detailing the required amendments. When these have been addressed, you’ll receive a formal acceptance letter and your manuscript will be scheduled for publication.

An invoice will be generated when your article is formally accepted. Please note, if your institution has a publishing partnership with PLOS and your article meets the relevant criteria, all or part of your publication costs will be covered. Please make sure your user information is up-to-date by logging into Editorial Manager at Editorial Manager® and clicking the ‘Update My Information' link at the top of the page. For questions related to billing, please contact billing support.

If your institution or institutions have a press office, please notify them about your upcoming paper to help maximize its impact. If they’ll be preparing press materials, please inform our press team as soon as possible -- no later than 48 hours after receiving the formal acceptance. Your manuscript will remain under strict press embargo until 2 pm Eastern Time on the date of publication. For more information, please contact onepress@plos.org.

Kind regards,

Emil George Haritinian, M.D, Ph.D.

Academic Editor

PLOS One

Additional Editor Comments (optional):

Reviewers' comments:

Reviewer's Responses to Questions

**Comments to the Author**

Reviewer #1: All comments have been addressed

Reviewer #2: All comments have been addressed

Reviewer #3: All comments have been addressed

**********

2. Is the manuscript technically sound, and do the data support the conclusions?

Reviewer #1: Yes

Reviewer #2: Yes

Reviewer #3: Yes

**********

3. Has the statistical analysis been performed appropriately and rigorously?

Reviewer #1: Yes

Reviewer #2: Yes

Reviewer #3: Yes

**********

4. Have the authors made all data underlying the findings in their manuscript fully available?

The PLOS Data policy

Reviewer #1: Yes

Reviewer #2: Yes

Reviewer #3: Yes

**********

5. Is the manuscript presented in an intelligible fashion and written in standard English?

Reviewer #1: Yes

Reviewer #2: Yes

Reviewer #3: Yes

**********

Reviewer #1: (No Response)

Reviewer #2: Thank you for your detailed responses to my comments. I appreciate the considerable effort made to revise the manuscript. The authors have adequately addressed my previous concerns by providing additional methodological details, clarifying the definitions, revising the statistical methods, and moderating the conclusions to reflect the limitations of the study. I have no further major comments and believe the manuscript is suitable for publication.

Reviewer #3: The author has thoroughly responded to the opinions and suggestions I raised and made corresponding revisions. Now the magazine has accepted and published this research.

**********

what does this mean?). If published, this will include your full peer review and any attached files.

If you choose “no”, your identity will remain anonymous but your review may still be made public.

**Do you want your identity to be public for this peer review?**  For information about this choice, including consent withdrawal, please see our Privacy Policy

Reviewer #1: No

Reviewer #2: No

Reviewer #3: No

**********

# Editor report: Acceptance letter

**DOI:** 10.1371/journal.pone.0356245.r004

**Authors:** Emil George Haritinian

PONE-D-26-16987R1

PLOS One

Dear Dr. Dou,

I'm pleased to inform you that your manuscript has been deemed suitable for publication in PLOS One. Congratulations! Your manuscript is now being handed over to our production team.

At this stage, our production department will prepare your paper for publication. This includes ensuring the following:

* All references, tables, and figures are properly cited

* All relevant supporting information is included in the manuscript submission,

* There are no issues that prevent the paper from being properly typeset

You will receive further instructions from the production team, including instructions on how to review your proof when it is ready. Please keep in mind that we are working through a large volume of accepted articles, so please give us a few days to review your paper and let you know the next and final steps.

Lastly, if your institution or institutions have a press office, please let them know about your upcoming paper now to help maximize its impact. If they'll be preparing press materials, please inform our press team within the next 48 hours. Your manuscript will remain under strict press embargo until 2 pm Eastern Time on the date of publication. For more information, please contact onepress@plos.org.

You will receive an invoice from PLOS for your publication fee after your manuscript has reached the completed accept phase. If you receive an email requesting payment before acceptance or for any other service, this may be a phishing scheme. Learn how to identify phishing emails and protect your accounts at https://explore.plos.org/phishing.

If we can help with anything else, please email us at customercare@plos.org.

Thank you for submitting your work to PLOS ONE and supporting open access.

Kind regards,

PLOS ONE Editorial Office Staff

on behalf of

Dr. Emil George Haritinian

Academic Editor

PLOS One
